# Fractals, Wealth Distributions, and Non-Euclidean Geometry: Quadrant Lorenz Curves

**DOI:** 10.3390/e28101085

**Published:** 2026-10-02

**Authors:** Iddo Eliazar

**Affiliations:** School of Chemistry, Tel Aviv University, Tel Aviv 6997801, Israel; eliazar@tauex.tau.ac.il

**Keywords:** power Lorenz curves, arc Lorenz curves, rich and poor socioeconomic fractality, double-Pareto tails, phase transitions, inequality indices, 02.50.-r (probability theory, stochastic processes, and statistics), 89.65.-s (social and economic systems)

## Abstract

Lorenz curves are a socioeconomic method of coding the statistics of positive-valued random variables. Standing at the confluence of fractals, wealth distributions, and non-Euclidean geometry, this paper presents quadrant Lorenz curves (QLCs): Lorenz curves whose shapes are that of the symmetric quarters of the *p*-norm unit circle. On the one hand, QLCs have a single parameter, and a clear geometric meaning. On the other hand, a comprehensive exploration establishes that QLCs display an assortment of socioeconomic and statistical behaviors. (1) They span a continuum of socioeconomic states ranging from perfect equality to perfect inequality, with three phase transitions along the continuum. (2) They display local rich fractality, local poor fractality, and double-Pareto tails. (3) They have inherent socioeconomic and statistical symmetries. The exploration includes a detailed socioeconomic-inequality analysis that illuminates QLCs from multiple inequality-indices ‘angles’. In a nutshell: QLCs manifest a novel class of ‘fractal’ statistical distributions, and the QLC features are unmatched by those of any other such class.

## 1. Introduction

Fractals are objects that are invariant with respect to the operations of ‘zooming in’ and ‘zooming out’ [1,2,3]. Due to their scale-invariance property, fractals assume center-stage roles in the sciences, e.g., physical sciences [4,5,6,7,8,9], biology and medicine [10,11,12,13], economics and finance [14,15,16], and mathematics [17,18,19,20,21].

The scale-invariance property of fractals is often determined by power laws [22,23,24,25,26]. In turn—and in the context of positive-valued random variables—the following question arises naturally: what are the fractal statistical distributions that are determined by power laws? This question turns out to have multiple answers.

Indeed, there are many perspectives via which statistical distributions are coded. In turn, infusing different perspectives with power-law structures yields different fractal statistics. Principal examples of such perspectives and of the resulting fractal statistics are the following [27].

1. Survival function and cumulative distribution function ↦ Pareto statistics and inverted Pareto statistics of ‘type I’ [28].
2. Forward and backward hazard rates [29,30] ↦ Weibull and Frechet statistics [31,32,33].
3. Log-Laplace transform ↦ Levy-stable statistics [34,35,36].

In all of the above examples the scale-invariance appears on a Poisson-process ‘bedrock level’ [37].

A socioeconomic perspective via which statistical distributions are coded is that of Lorenz curves [38,39,40,41]. Infusing the Lorenz-curves perspective with a power-law structure yields the aforementioned Pareto statistics [42]. In this perspective the scale-invariance is socioeconomic, and it manifests ‘rich fractality’ and ‘poor fractality’ [42].

Lorenz curves reside in the unit square, and their coordinates are *x* and *y*. In [42] a power-law structure was infused into the Lorenz curves algebraically: the two coordinates were set to be coupled by the power-law relation $y=x^{p}$, where *p* is a positive power.

This work re-visits the socioeconomic perspective, and does so with a ‘twist’. Here a power-law structure is infused into the Lorenz curves geometrically: the Lorenz coordinates are set to be coupled by a *p*-norm relation, where *p* is a positive power (as above).

Shifting from the Euclidean norm to the non-Euclidean *p*-norm, the unit circle changes from $x^{2}+y^{2}=1$ to ${\left|x\right|}^{p}+{\left|y\right|}^{p}=1$. This shift induces a marked rotational change. On the one hand, the Euclidean unit-circle is invariant with respect to rotation in any given angle. On the other hand, the non-Euclidean unit-circle (p≠2) is invariant with respect to rotation in specific angles: integer multiples of the angle π/2.

The rotational change gives rise to four symmetric quarters of unit circles, Euclidean and non-Euclidean alike. In turn, these quarters give rise to a special class of Lorenz curves: quadrant Lorenz curves (QLCs), whose shape are that of the quarters.

This class—which is parameterized by a single positive power *p*—is devised and explored here. The exploration is comprehensive, and key properties that are unveiled by the exploration are the following.

- QLCs span a continuum of socioeconomic states that range from the perfect-equality extreme to the perfect-inequality extreme.
- In the non-extreme states, QLCs display—jointly and simultaneously—local versions of rich fractality and poor fractality.
- In the non-extreme states, the QLCs’ wealth statistics display—jointly and simultaneously—left and right Pareto tails.
- QLCs and their wealth statistics have phase transitions at the following power thresholds: p=1/2, p=1, and p=2.
- QLCs and their wealth statistics have inherent socioeconomic and statistical symmetries.

The exploration also includes a detailed socioeconomic-inequality analysis of QLCs. Using a host of inequality indices [43,44,45,46,47,48]—each index gauging socioeconomic inequality from a different ‘angle’—a multi-faceted ‘picture’ of the socioeconomic inequality of QLCs is constructed.

Introducing and investigating QLCs, this paper presents a novel answer to the above question: what are the fractal statistical distributions that are determined by power laws? Devised by infusing Lorenz curves with a *p*-norm structure, QLCs emerge at the confluence of: fractals, wealth distributions, and non-Euclidean geometry.

On the one hand, QLCs are simple and transparent objects: they have a single parameter, and their Lorenz shapes have a clear geometric meaning. On the other hand, QLCs produce an assortment of socioeconomic and statistical features; to date, this assortment is unmatched by any other known answer to the above fractal-statistics question.

The paper is organized as follows. Section 2 reviews the quantitative modeling of wealth distributions in human societies. Section 3 addresses “power Lorenz curves”: Lorenz curves that are generated by the algebraic infusion of a power-law structure. And Section 4 addresses “arc Lorenz curves”: Lorenz curves that are arcs of Euclidean circles. ‘Standing on the shoulders’ of Section 3 and Section 4, Section 5 devises QLCs as an ‘amalgam’ of power Lorenz curves and of arc Lorenz curves. Thereafter, the paper carries on with a comprehensive investigation of QLCs: fractality and inequality (Section 6); wealth moments (Section 7); wealth density (Section 8); and alternative wealth sampling (Section 9).

The paper ‘wraps up’ with a conclusion (Section 10). In the context of the ‘region’ of double-Pareto statistics, the conclusion describes the QLC as a ‘city state’ that is a microcosm of the region and of the region’s ‘superpower’—a general diffusion framework for double-Pareto models. In this regional metaphor, while the region at large is well known and well studied, the QLC city-state is not; this paper is apparently the first to discover the city-state, and to investigate its microcosm role.

## 2. Modeling Wealth Distributions

This section reviews (tersely) the quantitative modeling of wealth distributions in human societies. The review is structured as follows: the notions of distribution functions and of Lorenz curves (Section 2.1); the Pietra-formulae linkages between distribution functions and Lorenz curves (Section 2.2); and the socioeconomic extremes of perfect equality and of perfect inequality (Section 2.3). The section concludes with a compact ‘toolbox’ (Section 2.4): a few Lorenz-based results which will be used along the paper.

### 2.1. Distribution Functions and Lorenz Curves

Consider a human society with members whose individual wealth takes values in the range $\left[w_{\min},w_{\max}\right]$. Specifically: the lower-bound of the members’ wealth values is $w_{\min}$; the upper-bound of the members’ wealth values is $w_{\max}$; and $0\leq w_{\min}<w_{\max}\leq \infty$.

The common statistical method of quantifying the distribution of wealth among the society members is via a pair of distribution functions. A cumulative distribution function, henceforth termed “bottom–up” distribution function: $F_{BU}\left(w\right)$ ($w_{\min}\leq w\leq w_{\max}$). And a tail distribution function, henceforth termed “top–down” distribution function: $F_{TD}\left(w\right)$ ($w_{\min}\leq w\leq w_{\max}$). The definitions of these functions are as follows.

- Bottom–up: $F_{BU}\left(w\right)$ is the proportion of the society’s members whose individual wealth is no larger than the value *w*.
- Top–down: $F_{TD}\left(w\right)$ is the proportion of the society’s members whose individual wealth is larger than the value *w*.

These definitions imply that the distribution functions yield values in the unit-interval range ($0\leq F_{BU}\left(w\right),F_{TD}\left(w\right)\leq 1$), and that they sum up to one ($F_{BU}\left(w\right)+F_{TD}\left(w\right)=1$). Also (in the wide sense), the bottom–up distribution function $F_{BU}\left(w\right)$ is monotone increasing, and the top–down distribution function $F_{TD}\left(w\right)$ is monotone decreasing.

Now, order the society members increasingly according to their wealth, i.e., from the poorest (the member with the smallest wealth) to the richest (the member with the largest wealth). The common socioeconomic method of quantifying the distribution of wealth among the society members is via a pair of Lorenz curves [49,50]: a ‘bottom–up’ Lorenz curve $y=L_{BU}\left(x\right)$ (0≤x≤1); and a ‘top–down Lorenz curve $y=L_{TD}\left(x\right)$ (0≤x≤1). The definitions of these functions are as follows.

- Bottom–up: the poorest 100x% of the society’s population hold, collectively, 100y% of the society’s overall wealth.
- Top–down: the richest 100x% of the society’s population hold, collectively, 100y% of the society’s overall wealth.

These definitions imply that the Lorenz curves have the following shapes [49,50]. Both curves are monotone increasing (in the wide sense) from zero ($L_{BU}\left(0\right)=L_{TD}\left(0\right)=0$) to one ($L_{BU}\left(1\right)=L_{TD}\left(1\right)=1$). And, in addition to the monotonicity: the bottom–up Lorenz curve $y=L_{BU}\left(x\right)$ is convex; and the top–down Lorenz curve $y=L_{TD}\left(x\right)$ is concave. Also, the definitions imply that the two Lorenz curve are coupled by the relation $L_{BU}\left(x\right)+L_{TD}\left(1-x\right)=1$.

### 2.2. Pietra’s Formulae

Consider the distribution functions, $F_{BU}\left(w\right)$ and $F_{TD}\left(w\right)$, to be strictly monotone. Then, these functions have well-defined inverse functions that are also strictly monotone.

Specifically, the inverse of the bottom–up distribution function is the bottom–up quantile function: $Q_{BU}\left(u\right):=F_{BU}^{-1}\left(u\right)$ (0≤u≤1), which is monotone increasing from the wealth lower-bound ($Q_{BU}\left(0\right)=w_{\min}$) to the wealth upper-bound ($Q_{BU}\left(1\right)=w_{\max}$). And, the inverse of the top–down distribution function is the top–down quantile function: $Q_{TD}\left(u\right):=F_{TD}^{-1}\left(u\right)$ (0≤u≤1), which is monotone decreasing from the wealth upper bound ($Q_{TD}\left(0\right)=w_{\max}$) to the wealth lower bound ($Q_{TD}\left(1\right)=w_{\min}$).

Pietra’s formulae represent the society’s Lorenz curves in terms of the society’s quantile functions [49,50]. Indeed, denote by μ the mean wealth of the society’s members (and consider this mean wealth to be positive). Then, the following integral representations hold.

- Bottom–up Pietra formula:
  (1) $$L_{BU}\left(x\right)=\frac{1}{\mu }\int_{0}^{x}Q_{BU}\left(u\right)du$$
  (0≤x≤1).
- Top–down Pietra formula:
  (2) $$L_{TD}\left(x\right)=\frac{1}{\mu }\int_{0}^{x}Q_{TD}\left(u\right)du$$
  (0≤x≤1).

Note that the strict monotonicity of the quantile functions implies strict convexity/concavity of the Lorenz curves. Specifically, the monotone increasing shape of the bottom–up quantile function $Q_{BU}\left(u\right)$ implies that: the bottom–up Lorenz of Equation (1) is strictly convex. And the monotone decreasing shape of the top–down quantile function $Q_{TD}\left(u\right)$ implies that the top–down Lorenz of Equation (2) is strictly concave.

### 2.3. Socioeconomic Extremes

Due to their shapes (described in Section 2.1), the Lorenz curves are bounded as follows. The bottom–up Lorenz curve $y=L_{BU}\left(x\right)$ is bounded from below by the value zero, and is bounded from above by the diagonal line y=x. And the top–down Lorenz curve $y=L_{TD}\left(x\right)$ is bounded from below by the diagonal line y=x, and is bounded from above by the value one. Namely,

(3) $$0\leq L_{BU}\left(x\right)\leq x\leq L_{TD}\left(x\right)\leq 1$$

(0≤x≤1). The bounds of the general Lorenz curves manifest two diametric socioeconomic extremes: perfect equality and perfect inequality.

The perfect-equality extreme manifests a ‘pure communism’ scenario in which all society members have exactly the same (positive) wealth. The perfect-equality extreme is characterized by the diagonal-line Lorenz curves: $L_{BU}\left(x\right)=x$ and $L_{TD}\left(x\right)=x$. Also, the perfect-equality extreme is the only case in which the two Lorenz curves coincide, $L_{BU}\left(x\right)=L_{TD}\left(x\right)$.

The perfect-inequality extreme is a ‘winners-take-it-all’ scenario in which: a zero fraction of the society’s population possesses all the society’s wealth. (This scenario is attainable when the society’s population grows infinitely large.) The perfect-inequality extreme is characterized by the following Lorenz curves: $L_{BU}\left(x\right)=0$ (where 0≤x<1), and $L_{TD}\left(x\right)=1$ (where 0<x≤1).

For 0<x<1, the Lorenz curves that characterize the socioeconomic extremes are the only ones that are linear. In other words, Lorenz curves that are strictly convex/concave characterize neither the perfect-equality extreme nor the perfect-inequality extreme.

Equation (3) describes a ‘geometric ordering’ of general Lorenz curves. In turn, this geometric ordering reflects a ‘socioeconomic ordering’ of human societies. Indeed, from a socioeconomic perspective, human societies reside between the perfect-equality extreme on the one hand, and the perfect-inequality extreme on the other hand.

### 2.4. Toolbox

As noted at the close of Section 2.2, the Lorenz curves of the Pietra formulae are strictly convex/concave. In turn, as noted in Section 2.3, such Lorenz curves characterize neither of the socioeconomic extremes. Thus, for wealth distributions that are neither perfectly equal nor perfectly unequal: the Pietra formulae yield representations of quantile functions in terms of Lorenz curves.

Indeed, differentiating Equations (1) and (2) yield the following quantile representations: $Q_{BU}\left(u\right)=\frac{1}{\mu }L_{BU}^{\prime }\left(u\right)$ (0≤u≤1); and $Q_{TD}\left(u\right)=\frac{1}{\mu }L_{TD}^{\prime }\left(u\right)$ (0≤u≤1). In turn, the quantile representations yield the following bound representations: the wealth lower-bound is $w_{\min}=\frac{1}{\mu }L_{BU}^{\prime }\left(0\right)=\frac{1}{\mu }L_{TD}^{\prime }\left(1\right)$; and the wealth upper-bound is $w_{\max}=\frac{1}{\mu }L_{BU}^{\prime }\left(1\right)=\frac{1}{\mu }L_{TD}^{\prime }\left(0\right)$.

With no loss of generality, set the mean wealth of the society’s members to be one: μ=1. Then—in terms of a general Lorenz curve $y=L\left(x\right)$ (be it bottom–up or top–down)—the above representations admit the following ‘unisex’ formulations.

- The quantile function (be it bottom–up or top–down) is the derivative of the Lorenz curve:
  (4) $$Q\left(u\right)=L^{\prime }\left(u\right)$$
  (0≤u≤1).
- The wealth bounds are the minimum and the maximum of the Lorenz-curve’s endpoint slopes:
  (5) $$w_{\min}=\min\left\{L^{\prime }\left(0\right),L^{\prime }\left(1\right)\right\}\;\&\;w_{\max}=\max\left\{L^{\prime }\left(0\right),L^{\prime }\left(1\right)\right\}\;.$$

Equations (4) and (5) are a compact ‘toolbox’ that will be used time and again in the sections below.

## 3. Power Lorenz Curves

Consider the power-law curve

(6) $$\varphi_{p}\left(x\right)=x^{p}$$

(0≤x≤1), where the power *p* is a positive parameter. The first and second derivatives of this curve are, respectively, $\varphi_{p}^{\prime }\left(x\right)=px^{p-1}$ and $\varphi_{p}^{\prime \prime }\left(x\right)=p\left(p-1\right)x^{p-2}$.

Arguably, the power-law curve of Equation (6) is one of the simplest examples of Lorenz curves. Indeed, this curve is monotone increasing ($\varphi_{p}^{\prime }\left(x\right)>0$ for 0<x<1) from zero ($\varphi_{p}\left(0\right)=0$) to one ($\varphi_{p}\left(1\right)=1$), and its Lorenz classification is as follows.

- When the power is smaller than one (0<p<1) the curve is strictly concave ($\varphi_{p}^{\prime \prime }\left(x\right)<0$), and hence it manifests a top–down Lorenz curve. In the limit p→0 the curve yields the top–down characterization of the perfect-inequality extreme: $\lim_{p\to 0}\varphi_{p}\left(x\right)=1$ (0<x≤1).
- When the power is one (p=1) the curve is the diagonal line ($\varphi_{p}\left(x\right)=x$), and hence it characterizes the perfect-inequality extreme.
- When the power is larger than one (1<p<∞) the curve is strictly convex ($\varphi_{p}^{\prime \prime }\left(x\right)>0$), and hence it manifests a bottom–up Lorenz curve. In the limit p→∞ the curve yields the bottom–up characterization of the perfect-inequality extreme: $\lim_{p\to \infty }\varphi_{p}\left(x\right)=0$ (0≤x<1).

**Figure 1:**
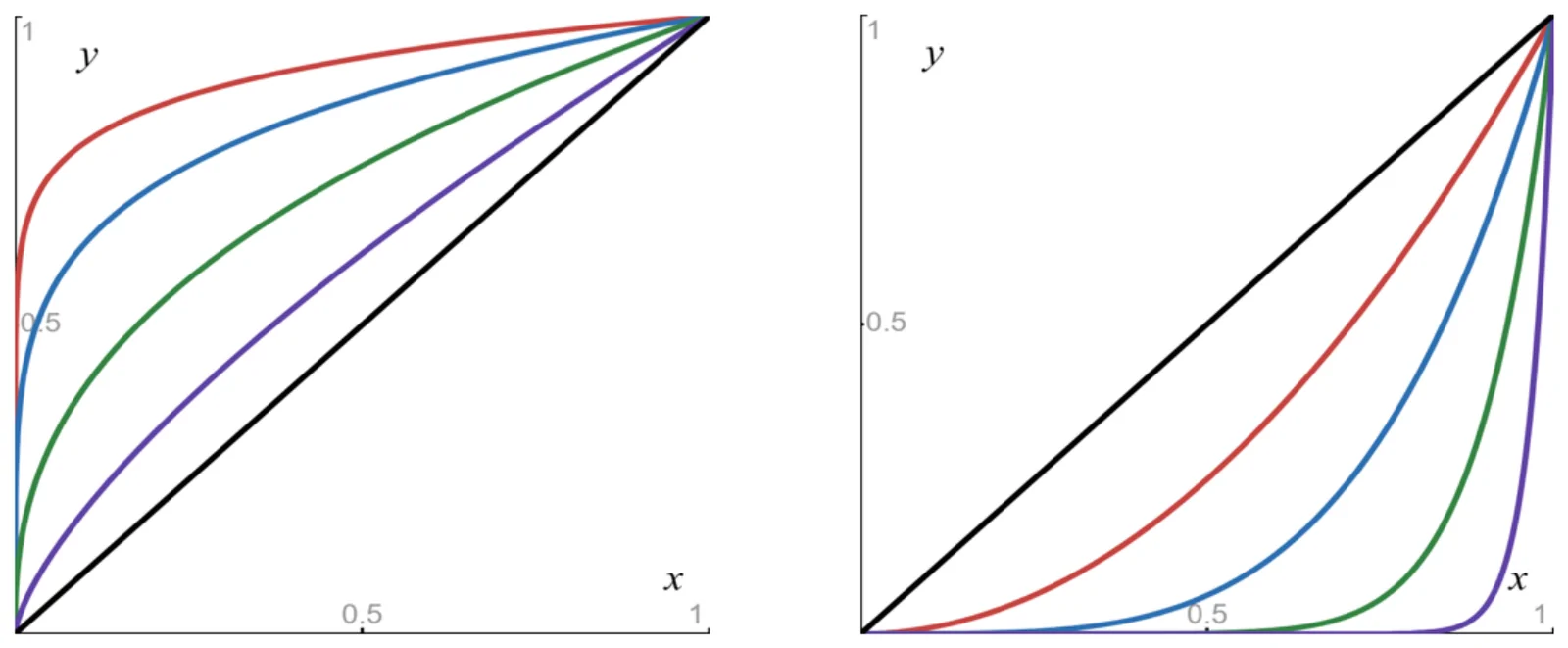
Examples of the power Lorenz curve of Equation (6). (**Left panel**) curves that correspond to the powers 0.1 (red), 0.2 (blue), 0.4 (green), 0.7 (purple), and 1 (black). (**Right panel**) curves that correspond to the powers 2 (red), 4 (blue), 10 (green), 30 (purple), and 1 (black).

The power-law curve of Equation (6) is henceforth termed “power Lorenz curve” (PLC). Examples of this curve are depicted in Figure 1. The PLC wealth statistics are addressed (Section 3.1) and discussed (Section 3.2) below.

### 3.1. Wealth Statistics

Consider the ‘non-diagonal’ cases (p≠1) of the PLC of Equation (6). Applying Equation (4) to the curve yields the quantile function $Q\left(u\right)=pu^{p-1}$ (0≤u≤1). In turn, inverting this quantile function yields the distribution function

(7) $$F\left(w\right)={\left(\frac{w}{p}\right)}^{1/(p-1)}$$

($w_{\min}\leq w\leq w_{\max}$). Yet in turn, Equation (5) yields the following classification of the distribution function of Equation (7).

- When the power is smaller than one (0<p<1) the distribution function is top–down, and its wealth bounds are $w_{\min}=p$ and $w_{\max}=\infty$.
- When the power is larger than one (1<p<∞) the distribution function is bottom–up, and its wealth bounds are $w_{\min}=0$ and $w_{\max}=p$.

In the top–down case (0<p<1) Equation (7) characterizes the ‘type I’ Pareto distribution [28], and it displays a right Pareto tail with exponent β=1/(1−p). Specifically, at infinitely large wealth values the asymptotics of the top–down distribution function are $F\left(w\right)∼1/w^{\beta }$ (as w→∞).

In the bottom–up case (1<p<∞) Equation (7) characterizes the inverted ‘type I’ Pareto distribution [28], and it displays a left Pareto tail with exponent α=1/(p−1). Specifically, at infinitesimally small wealth values the asymptotics of the bottom–up distribution function are $F\left(w\right)∼w^{\alpha }$ (as w→0).

### 3.2. Discussion

Key remarks regarding the PLC of Equation (6), and regarding the corresponding wealth statistics, are noted below.

**Fractal wealth distributions**. In the ‘non-diagonal’ cases (p≠1) the PLC of Equation (6) is of special importance. Indeed, the curve characterizes socioeconomic fractality [42]. Specifically, in the top–down case (0<p<1) the curve characterizes ‘rich fractality’ [42]: the unique socioeconomic state in which the distribution of wealth among the rich society members is identical to the distribution of wealth among all society members. And in the bottom–up case (1<p<∞) the curve characterizes ‘poor fractality’ [42]: the unique socioeconomic state in which the distribution of wealth among the poor society members is identical to the distribution of wealth among all society members. In both these fractal socioeconomic states the fractal exponent is the power *p* (of the PLC of Equation (6)).

**Phase transition and emergent wealth bounds**. The PLC of Equation (6) transits smoothly from its strictly concave shape (0<p<1) to its strictly convex shape (1<p<∞). Shifting from these Lorenz-curve cases to the corresponding distribution-function cases (of Equation (7)), the smooth transition of the former turns into a dramatic phase transition of the latter: from a statistical distribution over the infinite range [p,∞] to a statistical distribution over the finite range [0,p].

In this shift the power *p* emerges as a wealth bound. In the top–down case (0<p<1), and with regard to rich fractality: the power turns out to be the wealth lower bound, $w_{\min}=p$. In the bottom–up case (1<p<∞), and with regard to poor fractality: the power turns out to be the wealth upper bound, $w_{\max}=p$.

**Table 1 and Table 2**. Following the above remarks: a Lorenz classification and a socioeconomic classification of the PLC of Equation (6) are summarized in Table 1; and main statistical properties of the rich-fractality and the poor-fractality socioeconomic states are summarized in Table 2.

## 4. Arc Lorenz Curves

Section 3 addressed a specific class of Lorenz curves: power laws. This section addresses an altogether different class of Lorenz curves: arcs, namely, Lorenz curves that are arcs of circles [51]. Along this section the term “unit square” refers to the subset of the plane whose coordinates are 0≤x,y≤1.

Consider a circle that is centered at the point $\left(x_{0},y_{0}\right)$ of the plane, and whose radius is *r*. The circle points are given, implicitly, by the formula ${\left(x-x_{0}\right)}^{2}+{\left(y-y_{0}\right)}^{2}=r^{2}$. Lorenz curves pass through the points (0,0) and (1,1). Consequently, necessary conditions for Lorenz curves to be arcs are the following: the circle must pass through the points (0,0) and (1,1).

Specifically, the necessary conditions are ${x_{0}}^{2}+{y_{0}}^{2}=r^{2}$ and ${\left(1-x_{0}\right)}^{2}+{\left(1-y_{0}\right)}^{2}=r^{2}$. In turn, these necessary conditions imply that $x_{0}+y_{0}=1$. Namely, the circle’s center $\left(x_{0},y_{0}\right)$ must be located along the line $x_{0}+y_{0}=1$. There are three location scenarios.

One scenario is to place the circle’s center within the unit square, i.e., $0<x_{0},y_{0}<1$. In this scenario the circle has no arc that connects the points (0,0) and (1,1), and that resides (fully) in the unit square. Hence this scenario is not feasible. Two other scenarios are to place the circle’s center either ‘North-West’ of the unit square or ‘South-East’ to it. These scenarios turn out to be feasible indeed, and they are addressed (Section 4.1 and Section 4.2) and discussed (Section 4.3) below.

### 4.1. North-West Scenario

Place the circle’s center North-West to the unit square, i.e., $x_{0}\leq 0$ and $y_{0}\geq 0$. Set $x_{0}=-l$ where *l* is a non-negative location parameter, and note that $y_{0}=1+l$. In terms of this setting the circle formula is ${\left(x+l\right)}^{2}+{\left(y-1-l\right)}^{2}=r^{2}$. In turn, the fact that the circle passes through the points (0,0) and (1,1) implies that the circle’s squared radius is $r^{2}=l^{2}+{\left(1+l\right)}^{2}$.

The circle’s formula and radius yield the arc curve

(8) $$\psi_{l}\left(x\right)=\left(1+l\right)-\sqrt{r^{2}-{\left(l+x\right)}^{2}}$$

(0≤x≤1). The derivative of this curve is $\psi_{l}^{\prime }\left(x\right)=\left(l+x\right)/\sqrt{r^{2}-{\left(l+x\right)}^{2}}$.

Evidently, the arc curve of Equation (8) is monotone increasing ($\psi_{l}^{\prime }\left(x\right)>0$ for 0<x<1) from zero ($\psi_{l}\left(0\right)=0$) to one ($\psi_{l}\left(1\right)=1$). Also, the location of the circle’s center and the circle’s shape imply that the arc curve is strictly convex. So, Equation (8) manifests a bottom–up Lorenz curve.

Applying Equation (4) to the arc curve of Equation (8) yields the bottom–up quantile function $Q_{BU}\left(u\right)=\left(l+u\right)/\sqrt{r^{2}-{\left(l+u\right)}^{2}}$ (0≤u≤1). In turn, inverting this quantile function yields the bottom–up distribution function

(9) $$F_{BU}\left(w\right)=r\cdot \sqrt{\frac{w^{2}}{1+w^{2}}}-l$$

($w_{\min}\leq w\leq w_{\max}$). Yet in turn, Equation (5) yields the wealth bounds $w_{\min}=l/\left(1+l\right)$ and $w_{\max}=\left(1+l\right)/l$.

### 4.2. South-East Scenario

Place the circle’s center South-East to the unit square, i.e., $x_{0}\geq 1$ and $y_{0}\leq 0$. Set $x_{0}=1+l$ where *l* is a non-negative location parameter, and note that $y_{0}=-l$. In terms of this setting the circle formula is ${\left(x-1-l\right)}^{2}+{\left(y+l\right)}^{2}=r^{2}$. In turn, the fact that the circle passes through the points (0,0) and (1,1) implies that the circle’s squared radius is $r^{2}=l^{2}+{\left(1+l\right)}^{2}$.

The circle’s formula and radius yield the arc curve

(10) $${\bar{\psi }}_{l}\left(x\right)=\sqrt{r^{2}-{\left(1+l-x\right)}^{2}}-l$$

(0≤x≤1). The derivative of this curve is ${\bar{\psi }}_{l}^{\prime }\left(x\right)=\left(1+l-x\right)/\sqrt{r^{2}-{\left(1+l-x\right)}^{2}}$.

Evidently, the arc curve of Equation (10) is monotone increasing (${\bar{\psi }}_{l}^{\prime }\left(x\right)>0$ for 0<x<1) from zero (${\bar{\psi }}_{l}\left(x\right)\left(0\right)=0$) to one (${\bar{\psi }}_{l}\left(1\right)=1$). Also, the location of the circle’s center and the circle’s shape imply that the arc curve is strictly concave. So, Equation (10) manifests a top–down Lorenz curve.

Applying Equation (4) to the arc curve of Equation (10) yields the top–down quantile function $Q_{TD}\left(u\right)=\left(1+l-u\right)/\sqrt{r^{2}-{\left(1+l-u\right)}^{2}}$ (0≤u≤1). In turn, inverting this quantile function yields the top–down distribution function

(11) $$F_{TD}\left(w\right)=1+l-r\cdot \sqrt{\frac{w^{2}}{1+w^{2}}}$$

($w_{\min}\leq w\leq w_{\max}$). Yet in turn, the conclusion of Equation (5) yields the wealth bounds $w_{\min}=l/\left(1+l\right)$ and $w_{\max}=\left(1+l\right)/l$.

### 4.3. Discussion

Key remarks regarding the arc Lorenz curves of Equations (8) and (10), and regarding the corresponding wealth statistics, are noted below.

**Conjugation and diagonal symmetry**. In effect, the North-West scenario and the South-East scenario are conjugates of each other. Indeed, it follows from Equations (8) and (10) that $\psi_{l}\left(x\right)+{\bar{\psi }}_{l}\left(1-x\right)=1$ (0≤x≤1), and thus: the arc Lorenz curves are the conjugates of each other. This conjugation carries on to the above quantile functions, $Q_{BU}\left(u\right)=Q_{TD}\left(1-u\right)$, as well as to the distribution functions of Equations (9) and (11): $F_{BU}\left(w\right)+F_{TD}\left(w\right)=1$.

In addition to being the conjugates of each other, the arc Lorenz curves are the inverse functions of each other. Indeed, it follows from Equations (8) and (10) that the inverse function of the curve $y=\psi_{l}\left(x\right)$ is the curve $x={\bar{\psi }}_{l}\left(y\right)$, and the inverse function of the curve $y={\bar{\psi }}_{l}\left(x\right)$ is the curve $x=\psi_{l}\left(y\right)$. This inversion relationship manifests a ‘diagonal symmetry’ of the arc Lorenz curves: mirroring an arc Lorenz curve about the diagonal line y=x yields the conjugate arc Lorenz curve.

**Quadrant Lorenz curves**. The formulae presented above (in Section 4.1 and Section 4.2) imply that the following statements are equivalent.

1. The circle’s center is either at the North-West corner or the South-East corner of the unit square: $\left(x_{0},y_{0}\right)=\left(0,1\right)$ or $\left(x_{0},y_{0}\right)=\left(1,0\right)$.
2. The location parameter is zero: l=0.
3. The circle’s radius is one: r=1.
4. The wealth lower bound is zero: $w_{\min}=0$.
5. The wealth upper bound is infinity: $w_{\max}=\infty$.

Geometrically, these equivalent statements characterize the case in which the arc Lorenz curves are circle quadrants: quarters of the unit circle (i.e., a circle with radius one). Socioeconomically—regarding the wealth statistics of arc Lorenz curves—these equivalent statements characterize the case in which the wealth values are unbounded (i.e., the wealth values can be infinitesimally small as well as infinitely large).

## 5. Quadrant Lorenz Curves: Beyond Euclidean Geometry

Section 3 addressed power Lorenz curves. Addressing arc Lorenz curves, Section 4 concluded with quadrant Lorenz curves. These specific Lorenz curves have upsides (merits) and downsides (limitations).

An upside of power Lorenz curves is that they span a spectrum of socioeconomic states which range from the perfect-equality extreme to the perfect-inequality; moreover, these socioeconomic states have wealth statistics with Pareto tails. A downside of power Lorenz curves is the intrinsic wealth bounds of their wealth statistics.

An upside of quadrant Lorenz curves is that their corresponding wealth statistics have no intrinsic wealth bounds; moreover, these curves display a diagonal symmetry. A downside of quadrant Lorenz curves is that they manifest a single socioeconomic state, and hence they offer no span.

The goal of this section is to devise a class of Lorenz curves that combines the above upsides, and that circumvents the above downsides: on the one hand, as in the case of power Lorenz curves, a socioeconomic-span feature, and a Pareto-tails feature; on the other hand, as in the case of quadrant Lorenz curves, an unbounded-wealth feature, and a diagonal-symmetry feature.

This section’s goal will be accomplished via a route that goes beyond Euclidean geometry. The section is structured as follows: the ‘beyond Euclidean’ route (Section 5.1); the resulting quadrant Lorenz curves (Section 5.2); and the corresponding wealth statistics (Section 5.3). The section concludes with a summary (Section 5.4).

### 5.1. From Euclidean Circles to p-Norm Circles

Underlying the quadrant Lorenz curves is Euclidean geometry. Indeed, these Lorenz curves are quarters of the unit circle, and the unit circle is based on the underlying Euclidean norm: the Euclidean distance ${\left(x_{1}-x_{2}\right)}^{2}+{\left(y_{1}-y_{2}\right)}^{2}$ between any two points $\left(x_{1},y_{1}\right)$ and $\left(x_{2},y_{2}\right)$ of the plane.

This section will accomplish its goal by ‘elevating’ from the Euclidean norm to the, so-called, “*p*-norm”. In this norm the distance between two points $\left(x_{1},y_{1}\right)$ and $\left(x_{2},y_{2}\right)$ is: $|x_{1}-x_{2}{|}^{p}+{\left|y_{1}-y_{2}\right|}^{p}$, where the power *p* is a positive parameter. Evidently, the power p=2 generates the Euclidean norm. The power p=1 generates the, so-called, Manhattan norm.

The implicit formula of a *p*-norm circle that is centered at the point $\left(x_{0},y_{0}\right)$ of the plane, and whose radius is *r*, is

(12) $$|x-x_{0}{|}^{p}+{\left|y-y_{0}\right|}^{p}=r^{p}\;.$$

The four quadrants of the *p*-norm circle are (going clockwise) the following. North-East: $x\geq x_{0}$ and $y\geq y_{0}$. South-East: $x\geq x_{0}$ and $y\leq y_{0}$. South-West: $x\leq x_{0}$ and $y\leq y_{0}$. North-West: $x\leq x_{0}$ and $y\geq y_{0}$.

The upper quadrants are the North ones ($x\geq x_{0}$), and the lower quadrants are the South ones ($x\leq x_{0}$). The quadrants’ shapes are determined by the power *p* as follows. 

0<p<1: the North quadrants are strictly convex curves; and the South quadrants are strictly concave curves.

p=1: all quadrants are lines.

1<p<∞: the North quadrants are strictly concave curves; and the South quadrants are strictly convex curves. 

**Figure 2:**
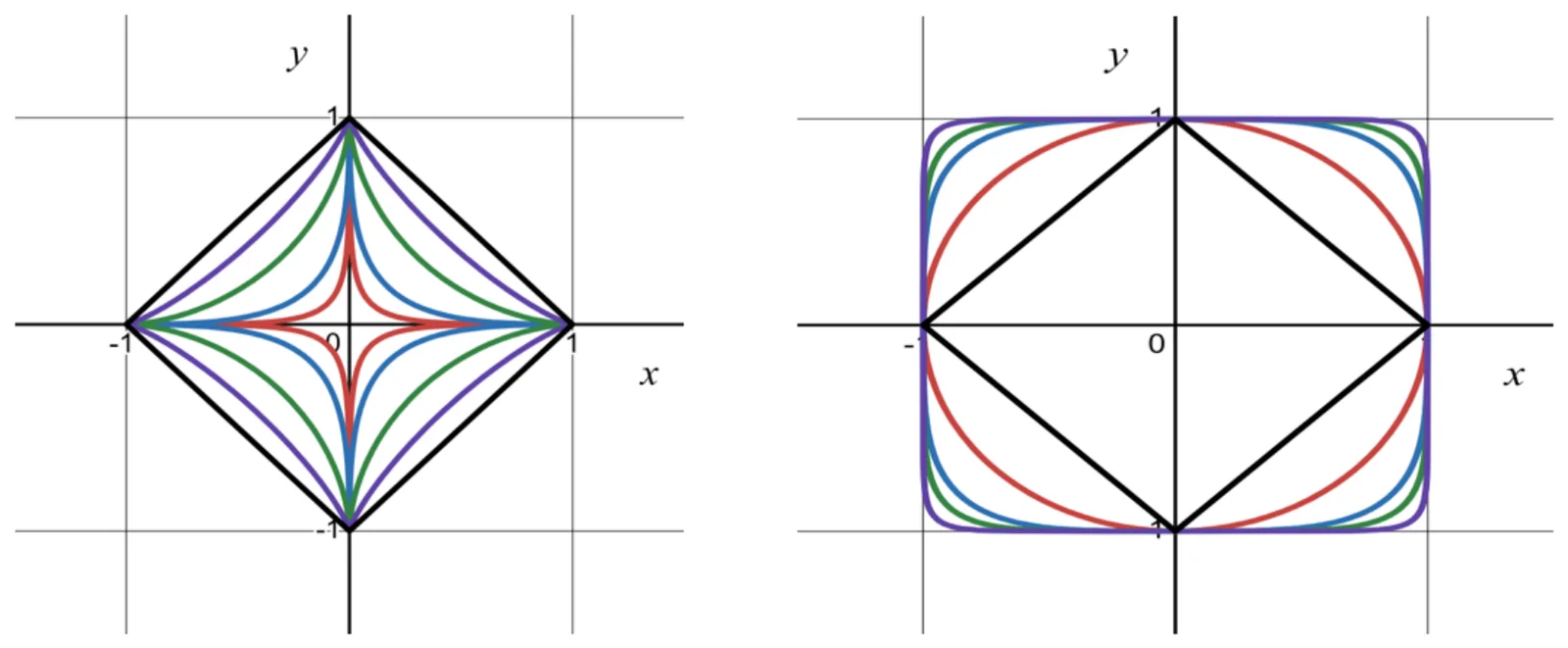
Examples of the p-norm circle of Equation (12), where the center is the origin of the plane, and the radius is one. (**Left panel**) circles that correspond to the powers 0.3 (red), 0.4 (blue), 0.6 (green), 0.8 (purple), and 1 (black). (**Right panel**) circles that correspond to the powers 2 (red), 4 (blue), 6 (green), 12 (purple), and 1 (black).

Examples of the *p*-norm circle of Equation (12) are depicted in Figure 2. In this figure: the circle is centered at the origin of the plane ($x_{0}=0$ and $y_{0}=0$); and the radius is one (r=1).

Along this section, as well as in the following sections, *q* denotes the ‘Holder conjugate’ of the power *p*. Namely, *p* and *q* are coupled by the Holder relation

(13) $$\frac{1}{p}+\frac{1}{q}=1\;.$$

In turn, q=p/(p−1) and hence: *q* is negative if and only if 0<p<1; and *q* is positive if and only if 1<p<∞.

### 5.2. Quadrant Lorenz Curves of *p*-Norm Circles

As in the Euclidean case of quadrant Lorenz curves (described in Section 4.3): circles with radius r=1 are placed at the North-West corner and at the South-East corner of the unit square. As described below, these *p*-norm circles yield a pair of quadrant curves.

Consider the circle whose center is placed at the North-West corner of the unit square $\left(x_{0},y_{0}\right)=\left(0,1\right)$. The circle’s South-East quadrant is the curve

(14) $$\phi_{p}\left(x\right)=1-{\left(1-x^{p}\right)}^{1/p}$$

(0≤x≤1). The derivative of this curve is $\phi_{p}^{\prime }\left(x\right)={\left(x^{-p}-1\right)}^{-1/q}$.

Evidently, the quadrant curve of Equation (14) is monotone increasing ($\phi_{p}^{\prime }\left(x\right)>0$ for 0<x<1) from zero ($\phi_{p}\left(0\right)=0$) to one ($\phi_{p}\left(1\right)=1$). Also, the location of the circle’s center and the circle’s shape yields the following conclusions regarding this curve.

- When the power is smaller than one (0<p<1) the curve is strictly concave, and hence it manifests a top–down Lorenz curve. In the limit p→0 the curve yields the top–down characterization of the perfect-inequality extreme: $\lim_{p\to 0}\phi_{p}\left(x\right)=1$ (0<x≤1).
- When the power is one (p=1) the curve is the diagonal line ($\phi_{p}\left(x\right)=x$), and hence it characterizes the perfect-equality extreme.
- When the power is larger than one (1<p<∞) the curve is strictly convex, and hence it manifests a bottom–up Lorenz curve. In the limit p→∞ the curve yields the bottom–up characterization of the perfect-inequality extreme: $\lim_{p\to \infty }\phi_{p}\left(x\right)=0$ (0≤x<1).

Consider now the circle whose center is placed at the South-East corner of the unit square $\left(x_{0},y_{0}\right)=\left(1,0\right)$. The circle’s North-West quadrant is the curve

(15) $${\bar{\phi }}_{p}\left(x\right)={\left[1-{\left(1-x\right)}^{p}\right]}^{1/p}$$

(0≤x≤1).

Equations (14) and (15) imply the coupling relation $\phi_{p}\left(x\right)+{\bar{\phi }}_{p}\left(1-x\right)=1$ (0≤x≤1). So, the quadrant curves of Equations (14) and (15) are conjugate Lorenz curves. The conjugate objects of Equations (14) and (15) are henceforth termed “quadrant Lorenz curves” (QLCs).

**Figure 3:**
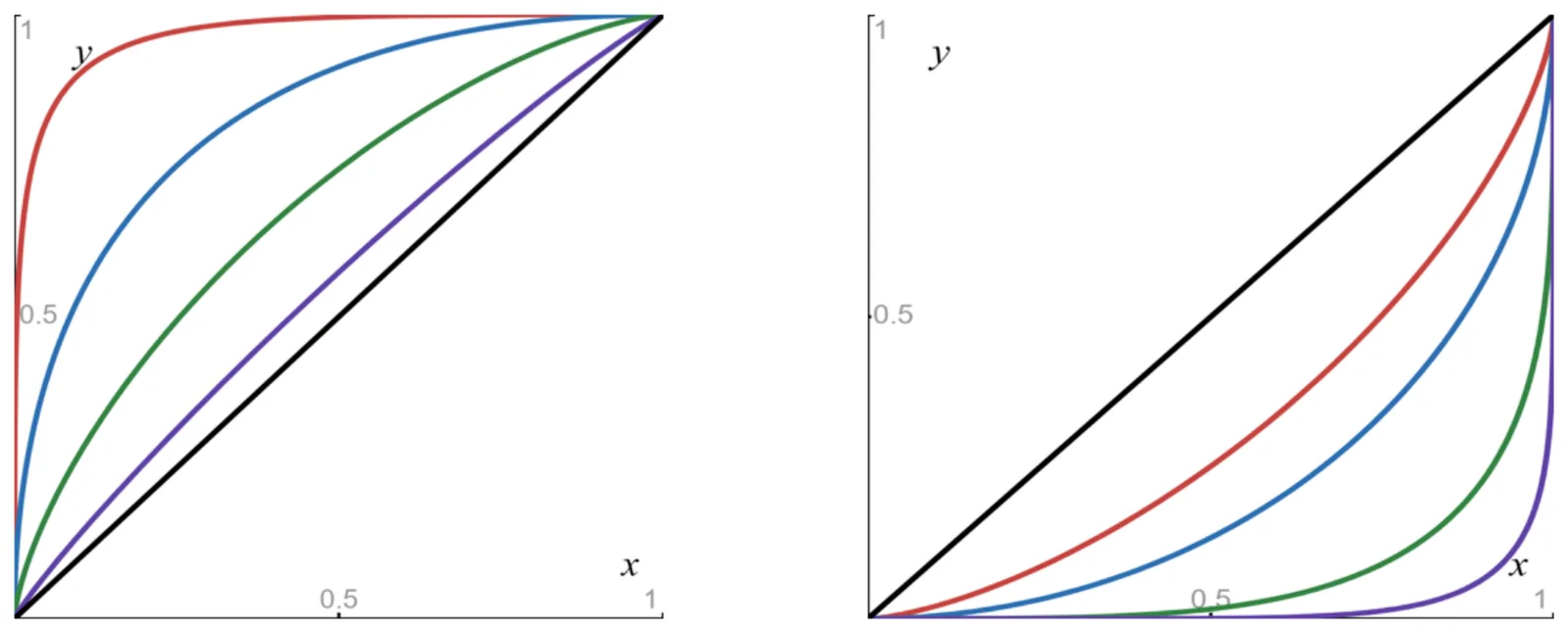
Examples of the quadrant Lorenz curve of Equation (14). (**Left panel**) curves that correspond to the powers 0.3 (red), 0.5 (blue), 0.7 (green), 0.9 (purple), and 1 (black). (**Right panel**) curves that correspond to the powers 1.5 (red), 2 (blue), 4 (green), 8 (purple), and 1 (black).

As noted in Section 5.1: the power p=1 generates the Manhattan norm; and the power p=2 generates the Euclidean norm. In turn, these powers yield the most basic examples of QLCs: the perfect-equality diagonal line (p=1); and the Euclidean QLC (p=2) of Section 4.3. Examples of the QLC of Equation (14) are depicted in Figure 3.

**Span feature**. The above shape conclusions regarding the QLC of Equation (14) describe a Lorenz classification. Note that this Lorenz classification is identical to that of the PLC of Equation (6). So, the QLCs tick the ‘span box’ of this section’s goal. Namely, as the PLCs, the QLCs span a spectrum of socioeconomic states which range from the perfect-equality extreme to the perfect-inequality extreme.

**Diagonal-symmetry feature**. In addition to being the conjugates of each other, the QLCs are the inverse functions of each other. Indeed, it follows from Equations (14) and (15) that: the inverse function of the curve $y=\phi_{p}\left(x\right)$ is the curve $x={\bar{\phi }}_{p}\left(y\right)$; and the inverse function of the curve $y={\bar{\phi }}_{p}\left(x\right)$ is the curve $x=\phi_{p}\left(y\right)$. So, the QLCs tick the ‘diagonal-symmetry box’ of this section’s goal. Namely, as in the case of arc Lorenz curves: mirroring a QLC about the diagonal line y=x yields the conjugate QLC.

### 5.3. Wealth Statistics

Consider the ‘non-diagonal’ cases (p≠1) of the QLCs. Applying Equation (4) to the QLC of Equation (14) yields the quantile function $Q\left(u\right)={\left(u^{-p}-1\right)}^{-1/q}$ (0≤u≤1). In turn, inverting this quantile function yields the distribution function

(16) $$F\left(w\right)=\frac{1}{{\left(1+w^{-q}\right)}^{1/p}}=\frac{w^{q/p}}{{\left(1+w^{q}\right)}^{1/p}}$$

($w_{\min}\leq w\leq w_{\max}$). Yet in turn, the conclusion of Equation (5) yields the wealth bounds $w_{\min}=0$ and $w_{\max}=\infty$.

When the power is smaller than one (0<p<1) then: the power’s Holder conjugate is negative (q<0), and the distribution function of Equation (16) is top–down. In this case the following Pareto tails are displayed.

- A left Pareto tail with exponent α=p/(1−p), i.e., at infinitesimally small wealth values the asymptotics are $1-F\left(w\right)∼$$w^{\alpha }$ (as w→0).
- A right Pareto tail with exponent β=1/(1−p), i.e., at infinitely large wealth values the asymptotics are $F\left(w\right)∼1/w^{\beta }$ (as w→∞).

When the power is larger than one (1<p<∞) then the power’s Holder conjugate is positive (q>0), and the distribution function of Equation (16) is bottom–up. In this case the following Pareto tails are displayed.

- A left Pareto tail with exponent α=1/(p−1), i.e., at infinitesimally small wealth values the asymptotics are $F\left(w\right)∼$$w^{\alpha }$ (as w→0).
- A right Pareto tail with exponent β=p/(p−1), i.e., at infinitely large wealth values the asymptotics are $1-F\left(w\right)∼1/w^{\beta }$ (as w→∞).

The Pareto exponents—for both the top–down and bottom–up cases—admit the following formulations. As functions of the positive power p≠1, the left and the right Pareto exponents are, respectively,

(17) $$\alpha \left(p\right)=\frac{\min\{p,1\}}{\left|p-1\right|}\;\;\;\&\;\;\;\beta \left(p\right)=\frac{\max\{p,1\}}{\left|p-1\right|}\;.$$

**Figure 4:**
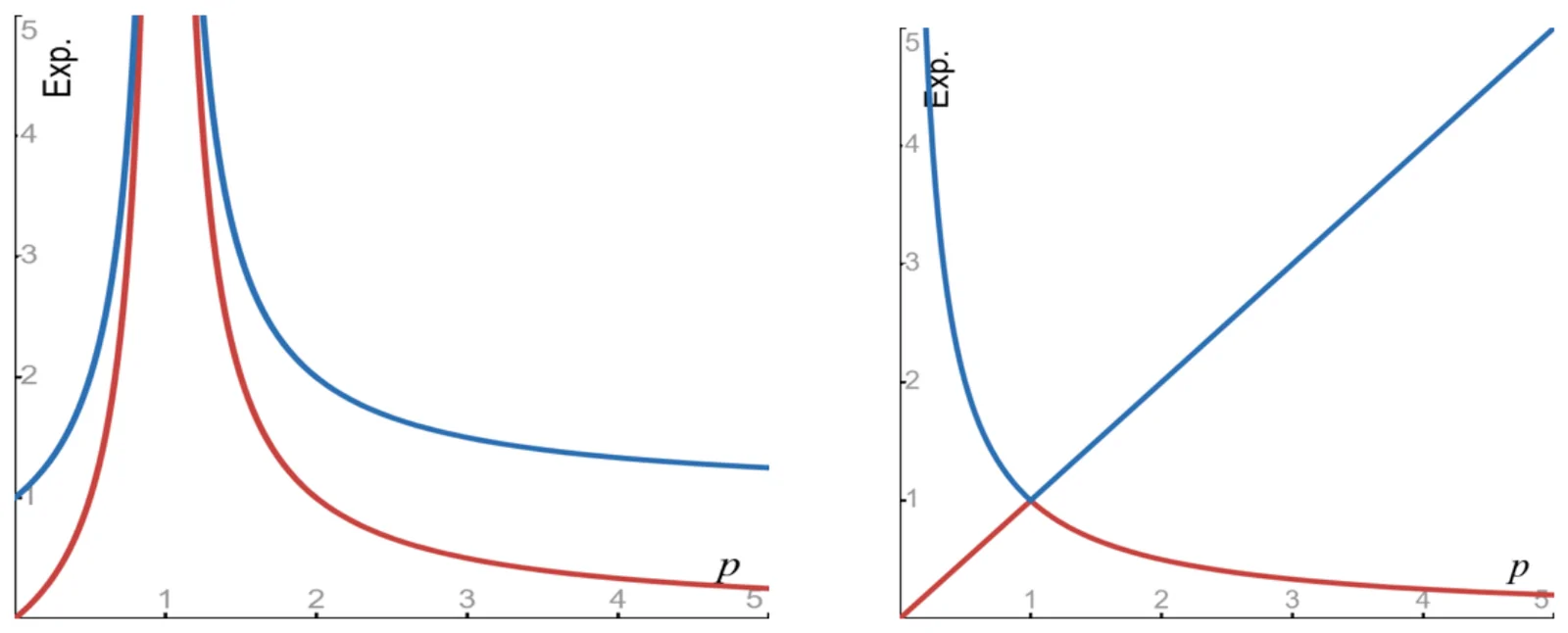
Pareto and fractality exponents as functions of the p-norm power. (**Left panel**) the left-tail Pareto exponent (red) and the right-tail Pareto exponent (blue) of Equation (17). (**Right panel**) the rich local-fractality exponent (red) and the poor local-fractality exponent (blue) of Equation (18).

The graphs of the Pareto exponents of Equation (17) are depicted in Figure 4. These graphs have unimodal shapes with singularities at p=1. Specifically, these graphs are: monotone increasing over the range 0<p<1 from $\lim_{p\to 0}\alpha \left(p\right)=0$ and $\lim_{p\to 0}\beta \left(p\right)=1$ to infinity ($\alpha \left(1\right)=\infty$ and $\beta \left(1\right)=\infty$); and monotone decreasing over the range 1<p<∞ from infinity ($\alpha \left(1\right)=\infty$ and $\beta \left(1\right)=\infty$) to $\lim_{p\to \infty }\alpha \left(p\right)=0$ and $\lim_{p\to \infty }\beta \left(p\right)=1$.

**Unbounded-wealth feature**. The above conclusion regarding the wealth bounds implies that the QLCs tick the ‘unbounded-wealth box’ of this section’s goal. Namely, as in the case of the Euclidean QLCs: the wealth values can be infinitesimally small as well as infinitely large.

**Pareto-tails feature**. The above conclusions regarding the Pareto tails imply that the QLCs tick the ‘Pareto-tails box’ of this section’s goal. Namely, the QLC wealth statistics display right and left Pareto tails that are identical—respectively—to the PLC right and left Pareto tails.

**Phase transition**. The QLC of Equation (14) transits smoothly from its strictly concave shape (0<p<1) to its strictly convex shape (1<p<∞). Shifting from these Lorenz-curve shapes to the corresponding distribution-function tails, the smooth transition of the former turns into a dramatic phase transition of the latter. Indeed, as evident from Equation (17): the QLC right and left Pareto tails change markedly as the power *p* crosses the perfect-equality threshold p=1.

### 5.4. Summary

This section set a goal of devising a class of Lorenz curves with four specified features. From a socioeconomic perspective: (1) span a spectrum of socioeconomic states that range from the perfect-equality extreme to the perfect-inequality extreme. From a Lorenz-curves perspective: (2) display a symmetry with respect to the diagonal line (which characterizes the perfect-equality extreme). And from a wealth-statistics perspective: (3) allow the wealth values to be unbounded; and (4) display left and right Pareto tails.

Euclidean quadrant Lorenz curves are quarters of Euclidean unit circles, they exhibit features #2 and #3, yet they exhibit neither feature #1 nor feature #4. Changing the underlying Euclidean norm to the *p*-norm, Euclidean circles ‘elevate’ to *p*-norm circles. Consequently, Euclidean quadrant Lorenz curves ‘elevate’ to *p*-norm quadrant Lorenz curves—termed “quadrant Lorenz curves” (QLCs) in short.

The elevation from the Euclidean norm to the *p*-norm infuses the QLCs with traits of PLCs. In turn, the resulting QLCs meet this section’s goal: as Euclidean QLCs—they exhibit features #2 and #3; going beyond Euclidean QLCs, and as PLCs—they further exhibit features #1 and #4.

Table 3 and Table 4 are counterparts, respectively, of Table 1 and Table 2 (which address the PLCs). Table 3 summarizes the Lorenz classification of the QLC of Equation (14), as well as the corresponding socioeconomic behaviors. Table 4 summarizes the main properties of the QLC wealth statistics (which are governed by the distribution function $F\left(w\right)$ of Equation (16)).

## 6. Quadrant Lorenz Curves: Fractality and Inequality

Section 5 established the QLCs. This section carries on with further exploration of these curves: their inherent fractality (Section 6.1), and their socioeconomic inequality (Section 6.2).

### 6.1. Fractality

Equations (14) and (15) imply the following asymptotics of the QLCs at their left endpoints: $\phi_{p}\left(x\right)∼x^{p}$ (as x→0); and ${\bar{\phi }}_{p}\left(x\right)$$∼x^{1/p}$ (as x→0). Namely (in the limit x→0): the QLC of Equation (14) is asymptotically equivalent to a PLC with power *p*; and the QLC of Equation (15) is asymptotically equivalent to a PLC with power 1/p. These asymptotics manifest a shift from ‘global’ fractality to ‘local’ fractality.

On the one hand, the fractality of the PLC of Equation (6) is global—manifesting scale invariance of the wealth statistics among the entire population. Specifically, when the power is smaller than one (0<p<1), then: zooming in from the wealth of all society members to the wealth of the rich members yields the very same wealth-statistics ‘picture’. And when the power is larger than one (1<p<∞) then: zooming in from the wealth of all society members to the wealth of the poor members yields the very same wealth-statistics ‘picture’.

On the other hand, the fractality of the QLCs is local—manifesting scale invariance of the wealth statistics among the rich and among the poor (rather than among the entire population). Specifically, zooming in from the wealth of the rich to the wealth of the super rich yields the very same wealth-statistics ‘picture’, and zooming in from the wealth of the *poor* to the wealth of the deep poor yields the very same wealth-statistics ‘picture’.

The global fractality of the PLC is either rich fractality or poor fractality, and its fractal exponent is the power *p*. Also, the global fractality has inherent wealth bounds: the wealth lower bound $w_{\min}=p$ in the case of rich fractality (0<p<1); and the wealth upper bound $w_{\max}=p$ in the case of poor fractality (1<p<∞). The local fractality of the QLCs has no such limitations: local rich fractality and local poor fractality appear jointly and simultaneously, and they have no inherent wealth bounds.

Indeed, in the shift from the PLC of Equation (6) to the QLCs of Equations (14) and (15) the following changes take place when the power is smaller than one (0<p<1). (1) Global rich fractality reduces to local rich fractality—both with fractal exponent *p*. (2) The wealth lower bound of global rich fractality ($w_{\min}=p$) goes away, and instead of this bound comes local poor fractality with fractal exponent 1/p.

And, in the shift from the PLC of Equation (6) to the QLCs of Equations (14) and (15) the following changes take place when the power is larger than one (1<p<∞). (1) Global poor fractality reduces to local poor fractality—both with fractal exponent *p*. (2) The wealth upper bound of global poor fractality ($w_{\max}=p$) goes away, and instead of this bound comes local rich fractality with fractal exponent 1/p.

The local-fractality exponents admit the following formulations. As functions of the positive power p≠1, the fractal exponents of local rich fractality and of local poor fractality are, respectively:

(18) $$\epsilon_{rich}\left(p\right)=\min\left\{p,\frac{1}{p}\right\}\;\;\;\&\;\;\;\epsilon_{poor}\left(p\right)=\max\left\{p,\frac{1}{p}\right\}\;.$$

Note that Equation (18) extends continuously to the power p=1—for which the local-fractality exponents are both one, $\epsilon_{rich}\left(1\right)=1$ and $\epsilon_{poor}\left(1\right)=1$.

As functions of the positive power *p*, the graphs of the local-fractality exponents $\epsilon_{rich}\left(p\right)$ and $\epsilon_{poor}\left(p\right)$ are depicted in Figure 4. The graph of the exponent $\epsilon_{rich}\left(p\right)$ has a unimodal shape that peaks at p=1, i.e., monotone increasing over the range 0<p≤1 from zero ($\lim_{p\to 0}\epsilon_{rich}\left(p\right)=0$) to one ($\epsilon_{rich}\left(1\right)=1$), and monotone decreasing over the range 1≤p<∞ from one ($\epsilon_{rich}\left(1\right)=1$) to zero ($\lim_{p\to \infty }\epsilon_{rich}\left(p\right)=0$). The graph of the exponent $\epsilon_{poor}\left(p\right)$ has a V shape that bottoms out at p=1, i.e., monotone decreasing over the range 0<p≤1 from infinity ($\lim_{p\to 0}$$\epsilon_{poor}\left(p\right)=\infty$) to one ($\epsilon_{poor}\left(1\right)=1$), and monotone increasing over the range 1≤p<∞ from one ($\epsilon_{poor}\left(1\right)=1$) to infinity ($\lim_{p\to \infty }\epsilon_{poor}\left(p\right)=\infty$).

The global fractality of the PLC of Equation (6) is summarized in the right column of Table 1, and the local fractality of the QLC of Equation (14) is summarized in the right column of Table 3. In turn, the shift from the global fractality of the former to the local fractality of the latter is summarized by: the shift from right column of Table 1 to the right column of Table 3.

**Phase transition**. Section 5.3 described a QLC phase transition that takes place as the power *p* crosses the perfect-equality threshold p=1. Section 5.3 illuminated the phase transition from a Pareto-tails perspective. This subsection illuminates the phase transition from a local-fractality perspective. Indeed, this subsection describes how the QLC local fractality—rich and poor alike—changes markedly as the power *p* crosses the threshold p=1.

### 6.2. Socioeconomic Inequality

An inequality index *I* [43,44,45,46,47,48] is a functional of Lorenz curves that satisfies the three following conditions. (1) It takes values in the range 0≤I≤1. (2) It is zero, I=0, only in the perfect-equality extreme. (3) In the perfect-inequality extreme it is one, I=1. With regard to a human society of interest: a given inequality index *I* provides a quantitative score for the degree of the society’s socioeconomic inequality [43,44,45,46,47,48].

There are many inequality indices, and hence there are many ways of scoring socioeconomic inequality. Several specific inequality indices are described in this subsection, and are applied to the QLC of Equation (14).

**Gini index**. Arguably, the best-known and the most widely applied inequality index is the Gini index $I_{Gini}$ [52,53,54,55,56,57,58]; this index has many interpretations and representations [47,54,58]. In terms of the Lorenz curve $y=L\left(x\right)$, the Gini index is twice the area captured between the Lorenz curve $y=L\left(x\right)$ and the diagonal line y=x, i.e., $I_{Gini}=2\int_{0}^{1}\left|L(x)-x\right|dx$.

Applying the Gini index to the QLC of Equation (14), an integral calculation (which uses the Beta function) yields the following result. As a function of the positive power *p*, the QLC Gini index is

(19) $$I_{Gini}\left(p\right)=\left|2\frac{\Gamma {\left(1+\frac{1}{p}\right)}^{2}}{\Gamma \left(1+\frac{2}{p}\right)}-1\right|\;.$$

The general properties of the Gini index, together with properties of the QLC of Equation (14), imply that the graph of the Gini index of Equation (19) has a V shape. Specifically, this graph is monotone decreasing over the range 0<p≤1 from one ($\lim_{p\to 0}I_{Gini}\left(p\right)=1$) to zero ($I_{Gini}\left(1\right)=0$), and monotone increasing over the range 1≤p<∞ from zero ($I_{Gini}\left(1\right)=0$) to one ($\lim_{p\to \infty }I_{Gini}\left(p\right)=1$). This shape is in accord with the socioeconomic behaviors of the QLCs (summarized in Table 3).

**Pietra index**. Another well-established inequality index is the Pietra index $I_{Pietra}$ [59,60,61,62,63,64]; also this index has many interpretations and representations [47]. In terms of the Lorenz curve $y=L\left(x\right)$, the Pietra index admits the following formulations. It is the maximal vertical distance between the Lorenz curve $y=L\left(x\right)$ and the diagonal line y=x, i.e., $I_{Pietra}=\max_{0\leq x\leq 1}\left|L\left(x\right)-x\right|$. And it is the maximal horizontal distance between the Lorenz curve $y=L\left(x\right)$ and the diagonal line y=x, i.e., $I_{Pietra}=\max_{0\leq y\leq 1}\left|L^{-1}\left(y\right)-y\right|$.

Applying the Pietra index to the QLC of Equation (14), a maximization calculation yields the following result. As a function of the positive power *p*, the QLC Pietra index is

(20) $$I_{Pietra}\left(p\right)=\left|2^{(p-1)/p}-1\right|\;.$$

**Figure 5:**
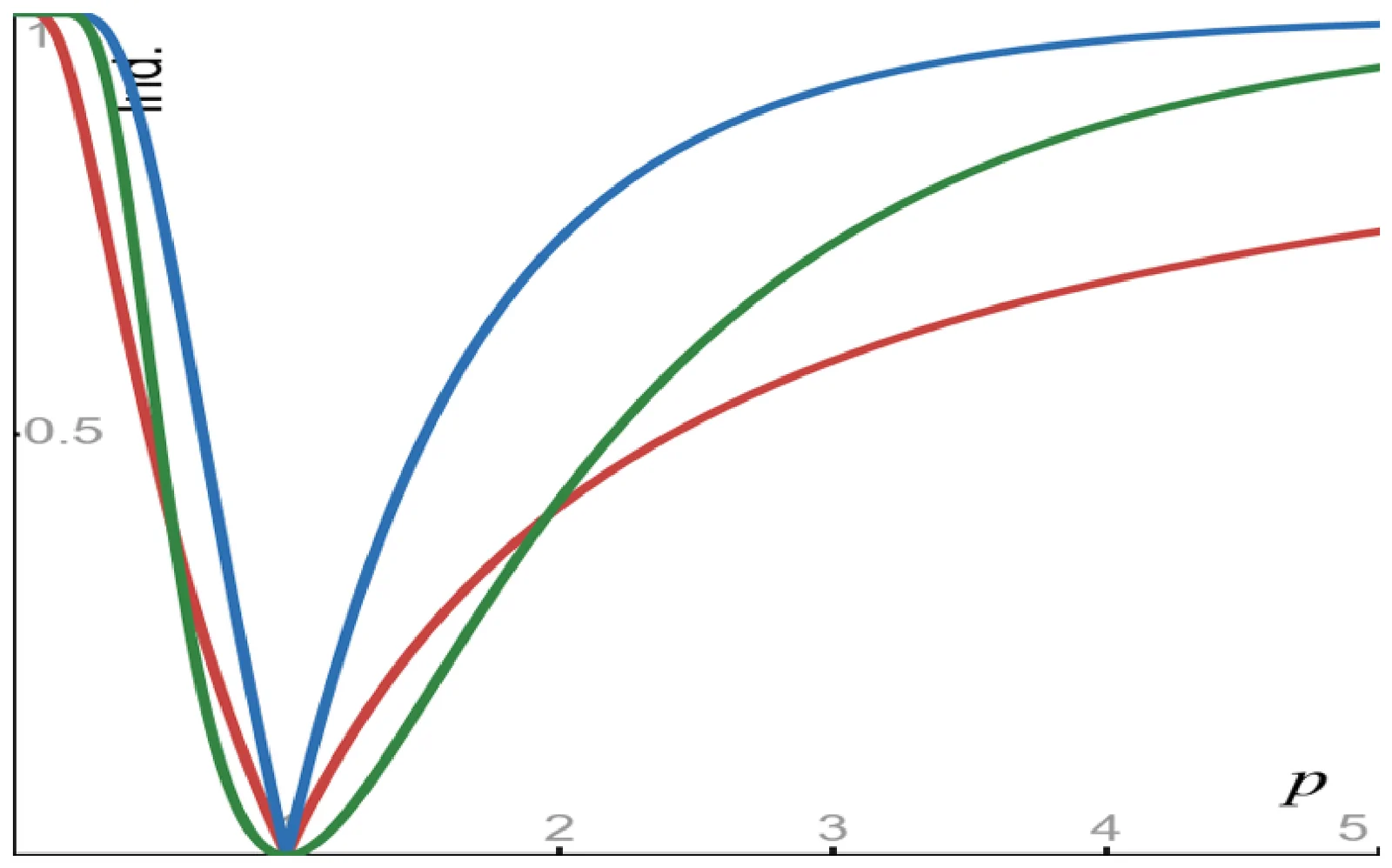
Inequality indices as functions of the p-norm power. The Pietra inequality index of Equation (20) (red), the diameter inequality index of Equation (21) (blue), and the median inequality index of Equation (30) (green).

The graph of the Pietra index of Equation (20) is depicted in Figure 5. The shape of this graph is the same as that of the Gini index of Equation (19).

**Kolkata index**. The line x+y=1 is perpendicular to the diagonal line y=x, and these two lines intersect at the ‘center’ of the unit square (0≤x,y≤1): the point $\left(\frac{1}{2},\frac{1}{2}\right)$. Set $\left(x_{*},y_{*}\right)$ to be the point at which the Lorenz curve $y=L\left(x\right)$ and the line x+y=1 intersect. Inspired by Pareto’s 20–80 principle, the Kolkata inequality index is the quantity $|x_{*}-\frac{1}{2}\left|=\right|y_{*}-\frac{1}{2}|$ [65,66,67,68]; also this index has several interpretations [47,49,50]. Applying this inequality index to the QLC of Equation (14) yields the same value as the QLC Pietra index of Equation (20).

**Diameter index**. Given a Lorenz curve $y=L\left(x\right)$, set $y=\bar{L}\left(x\right)$ to be its conjugate. (Namely, the two Lorenz curves are coupled by the relation $L\left(x\right)+\bar{L}\left(1-x\right)=1$ (0≤x≤1)). Following the ‘gist’ of the Pietra index: the vertical-diameter index [49,50] is the maximal vertical distance between the two Lorenz curves, $I_{Vdiam}=\max_{0\leq x\leq 1}\left|L\left(x\right)-\bar{L}\left(x\right)\right|$; and the horizontal-diameter index [49,50] is the maximal horizontal distance between the two curves, $I_{Hdiam}=\max_{0\leq y\leq 1}\left|L^{-1}\left(y\right)-{\bar{L}}^{-1}\left(y\right)\right|$. Both these maxima are attained at the point half, i.e., at $x=\frac{1}{2}$ in the case of $I_{Vdiam}$, and at $y=\frac{1}{2}$ in the case of $I_{Hdiam}$.

Due to their diagonal-symmetry feature, the top–down and bottom–up QLCs are the inverse functions of each other: $L^{-1}\left(y\right)=\bar{L}\left(y\right)$ and ${\bar{L}}^{-1}\left(y\right)=L\left(y\right)$. Consequently, the QLC vertical-diameter index and the QLC horizontal-diameter index coincide and yield a single diameter index: $I_{Diam}:=I_{Vdiam}=I_{Hdiam}$.

Setting $x=\frac{1}{2}$ in the QLC of Equation (14) (and using the coupling $L\left(\frac{1}{2}\right)+\bar{L}\left(\frac{1}{2}\right)=1$) yields the following result. As a function of the positive power *p*, the QLC diameter index is

(21) $$I_{Diam}\left(p\right)=\left|{\left(2^{p}-1\right)}^{1/p}-1\right|\;.$$

The graph of the diameter index of Equation (21) is depicted in Figure 5. The shape of this graph is the same as that of the Gini index of Equation (19).

## 7. Quadrant Lorenz Curves: Wealth Moments

Section 5.3 addressed the QLC wealth statistics. Carrying on from Section 5.3, this section explores the QLC moments, i.e., the moments of the QLC wealth statistics.

### 7.1. Lomax Representation and Wealth Moments

Set *W* to be a QLC random variable, i.e., a random variable whose statistics are governed by the distribution function $F\left(w\right)$ of Equation (16). Namely, when the power *p* is smaller than one (0<p<1) then $F\left(w\right)=\mathrm{Pr}\left(W>w\right)$. And when the power *p* is larger than one (1<p<∞) then $F\left(w\right)=\mathrm{Pr}\left(W\leq w\right)$.

The socioeconomic interpretation of the QLC random variable *W* is the following. Consider a human society whose Lorenz curve is that of Equation (14), and sample at random a single society member. Then, the random variable *W* manifests the wealth of the randomly sampled member.

The following observations hold with regard to the random variable $W_{*}=1/W^{q}$. When the power *p* is smaller than one then its Holder conjugate *q* is negative, and hence: $W_{*}>w$ if and only if $W>w^{-1/q}$. And when the power *p* is larger than one then its Holder conjugate *q* is positive, and hence: $W_{*}>w$ if and only if $W<w^{-1/q}$.

Combining the above observations with the distribution function of Equation (16) yields the following conclusion. The top–down distribution function of the random variable $W_{*}$ is

(22) $$\mathrm{Pr}\left(W_{*}>w\right)=\frac{1}{{\left(1+w\right)}^{1/p}}$$

(0≤w≤∞).

Equation (22) characterizes a Lomax—a.k.a. “Pareto type II” [28]—random variable with the following parameters: scale one and exponent 1/p. So, in terms of the Lomax random variable $W_{*}$, the QLC random variable *W* admits the ‘Lomax representation’ $W=W_{*}^{-1/q}$.

Differentiating the top–down Lomax distribution function of Equation (22) yields the Lomax density function: $f_{*}\left(w\right)=\frac{1}{p}{\left(1+w\right)}^{-(1+p)/p}$ (0≤w≤∞). In turn, an integral calculation (which uses the Beta function) yields the Lomax moments: $E\left[W_{*}^{m}\right]=\int_{0}^{\infty }w^{m}f_{*}\left(w\right)dw$ (−∞<m<∞). Specifically, the Lomax moment of order *m* converges if and only if $-1<m<\frac{1}{p}$, in which case it is $E\left[W_{*}^{m}\right]=\Gamma \left(1+m\right)\Gamma \left(\frac{1}{p}-m\right)/\Gamma \left(\frac{1}{p}\right)$.

The Lomax representation $W=W_{*}^{-1/q}$ implies the moments representation $E\left[W^{m}\right]=E\left[W_{*}^{-m/q}\right]$, i.e., a representation of the QLC moments in terms of the Lomax moments. In turn, the above Lomax-moments result yield the following QLC moments result. The QLC moment of order *m* converges if and only if −1<m(p−1)<p, in which case it is

(23) $$E\left[W^{m}\right]=\frac{\Gamma \left(1-\frac{m}{q}\right)\Gamma \left(\frac{1}{p}+\frac{m}{q}\right)}{\Gamma \left(\frac{1}{p}\right)}\;.$$

Note that when p=1 then $\frac{1}{q}=0$ and hence: setting p=1 in Equation (23) yields unit moments, $E[W^{m}]=1$ (−∞<m<∞). This implication, as well as other implications that follow from the QLC moments, are addressed below.

### 7.2. Moments Discussion

Section 7.1 concluded with the QLC moments—the moments of the QLC random variable *W*. Remarks regarding the QLC moments are noted in this subsection.

**Mean and variance**. The first and second QLC moments are, respectively, $E[W^{1}]$ and $E[W^{2}]$. The QLC mean is the first moment: $E\left[W\right]=E\left[W^{1}\right]$. And, the QLC variance is the difference between the second moment and the squared first moment: $\mathrm{Var}\left[W\right]=E\left[W^{2}\right]-E{\left[W\right]}^{2}$.

Setting m=1 in Equation (23) implies that the QLC mean is one, E[W]=1. And indeed, the mean was set to be one already in the toolbox of Section 2.4. Setting m=2 in Equation (23) yields the following variance result. The QLC variance is finite if and only if $\frac{1}{2}<p<2$, in which case it is

(24) $$\mathrm{Var}\left[W\right]=\frac{\Gamma \left(\frac{2}{p}-1\right)\Gamma \left(2-\frac{1}{p}\right)}{\Gamma \left(\frac{1}{p}\right)}-1\;.$$

Note that setting p=1 in Equation (24) yields a zero variance. This implication is in accord with the unit-moments implication (which was noted after Equation (23)), and both these implications are intimately related to the next remark.

**Perfect equality**. As explained in Section 5, and as summarized in Table 3, the QLC of Equation (14) yields the socioeconomic state of perfect equality when—and only when—its power is one (p=1). As the mean was set to be one, the perfect-equality state is characterized by a deterministic QLC random variable that is one: Pr(W=1)=1. Indeed, as all the society members have personal wealth one, sampling at random a single member of the society will always result in the wealth outcome W=1. Thus, setting p=1 in Equations (23) and (24) yields, respectively, the above implications: the unit-moments implication, and the zero-variance implication.

**Moments’ convexity symmetry**. Measuring the QLC random variable *W* via the gauge function $g\left(w\right)=w^{m}$ (0≤w≤∞) yields the measurement $g\left(W\right)=W^{m}$. In effect, the QLC moments are the mean of the measurement: $E\left[g\left(W\right)\right]=E\left[W^{m}\right]$. Note that the gauge function $g\left(w\right)=w^{m}$ is strictly convex when *m* is either in the range m<0, or in the range m>1.

Using the Holder relation of Equation (13), it follows from Equation (23) that

(25) $$E\left[W^{-m}\right]=E\left[W^{m+1}\right]$$

(−∞<m<∞). Consequently, when the parameter *m* is positive then Equation (25) manifests an intrinsic symmetry between the two ‘strictly convex ranges’ of the QLC moments. Indeed, −m<0 if and only if m+1>1.

In particular, the specific parameter value m=1 yields the following result. The mean of the reciprocal of the QLC random variable is equal to the second moment of the QLC random variable: $E\left[1/W\right]=E\left[W^{2}\right]$.

**Moments’ inequality indices**. Consider the mean measurement $E[g\left(W\right)]$, where g(w) (0≤w≤∞) is a gauge function with the following properties: it is non-negative; it is strictly convex; and it passes through the point g(1)=1. In turn, also the mean measurement is non-negative: $E[g\left(W\right)]\geq 0$. Moreover, Jensen’s inequality implies that: $E[g\left(W\right)]\geq 1$, with equality holding only when the QLC random variable is deterministic (Pr(W=1)=1).

Due to the above gauge-function properties, the quantity $1-1/E[g\left(W\right)]$ is an inequality index [69]. (The notion of inequality indices was defined in the opening of Section 6.2). In its ‘strictly convex ranges’, the gauge function $g\left(w\right)=w^{m}$ displays the above properties. Consequently, Equation (25) yields a continuum of moments inequality indices [47,69,70]: $I_{m}\left(p\right)$ (0<m<∞). Specifically, as functions of the positive power *p*, the moments inequality indices admit the following formulations: the ‘negative formulation’ $I_{m}\left(p\right)=1-1/E\left[W^{-m}\right]$; and the ‘positive formulation’ $I_{m}\left(p\right)=1-1/E\left[W^{m+1}\right]$.

**Variance inequality index**. In particular, the moments inequality indices include the following variance inequality index [47]: $I_{Var}\left(p\right)=\mathrm{Var}\left[W\right]/\left\{1+\mathrm{Var}\left[W\right]\right\}$. Indeed, setting m=1 in the ‘positive formulation’ of the moments inequality indices, and using the aforementioned facts regarding the QLC mean and the QLC variance (namely, E[W]=1 and $\mathrm{Var}\left[W\right]=E\left[W^{2}\right]-1$), yields the variance inequality index. (The QLC variance Var[W] is specified in Equation (24) above).

## 8. Quadrant Lorenz Curves: Wealth Density

Section 5.3 addressed the QLC wealth statistics, and Section 7.1 introduced the QLC random variable *W*. Carrying on from Section 5.3, this section explores the QLC wealth density, i.e., the probability density function—denoted $f\left(w\right)$ (0≤w≤∞)—of the QLC random variable *W*. The section begins with the QLC wealth density (Section 8.1), and then it continues with a couple of discussions (Section 8.2 and Section 8.3).

### 8.1. Wealth Density

As explained in Section 7.2, when p=1 then the QLC random variable is one: Pr(W=1)=1. In turn, when p=1 then the QLC wealth density is a Dirac ‘delta function’ that is centered at the point w=1.

When p≠1 then the statistics of the QLC random variable *W* are governed by the distribution function of Equation (16). Differentiating this distribution function implies that the wealth density is: $f\left(w\right)=-F^{\prime }\left(w\right)$ when the power is smaller than one (0<p<1); and $f\left(w\right)=F^{\prime }\left(w\right)$ when the power is larger than one (1<p<∞).

So, when p≠1 then the QLC wealth density is

(26) $$f\left(w\right)=\frac{\left|q\right|}{p}\frac{w^{-q-1}}{{\left(1+w^{-q}\right)}^{(1/p)+1}}=\frac{\left|q\right|}{p}\frac{w^{(q/p)-1}}{{\left(1+w^{q}\right)}^{(1/p)+1}}$$

(0<w<∞). The left and right endpoint values of the wealth density of Equation (26) are denoted, respectively: $f\left(0\right)=\lim_{w\to 0}f\left(w\right)$ and $f\left(\infty \right)=\lim_{w\to \infty }f\left(w\right)$.

At infinitely large wealth values (w→∞) the wealth density of Equation (26) decays to zero, $f\left(\infty \right)=0$. Depending on the power *p*, at infinitesimally small wealth values (w→0) the wealth density of Equation (26) displays markedly different behaviors.

- When the power is either smaller than half ($0<p<\frac{1}{2}$) or larger than two (2<p<∞) then the wealth density is infinite, $f\left(0\right)=\infty$.
- When the power is either half ($p=\frac{1}{2}$) or two (p=2) then then the wealth density is positive: $f\left(0\right)=2$ and $f\left(0\right)=1$, respectively.
- When the power is larger than half and smaller than two ($\frac{1}{2}<p<2$) then the wealth density is zero, $f\left(0\right)=0$.

Depending on the power *p*, the wealth density of Equation (26) displays markedly different shapes. To describe the shapes denote by $w_{mod}$ the wealth-density’s mode. Namely, $w_{mod}$ $=\arg\max_{0\leq w\leq \infty }f\left(w\right)$ is the point at which the wealth density peaks.

- When the power is either no-larger than half ($0<p\leq \frac{1}{2}$) or no-smaller than two (2≤p<∞) then the mode is zero ($w_{mod}=0$), and the wealth density is monotone decreasing over its entire range [0,∞].
- When the power is larger than half and smaller than two ($\frac{1}{2}<p<2$) then the mode is positive ($0<w_{mod}<\infty$), and the wealth density is unimodal—i.e., it is monotone increasing over the interval $\left[0,w_{mod}\right]$, and it is monotone decreasing over the ray $\left[w_{mod},\infty \right]$.

**Figure 6:**
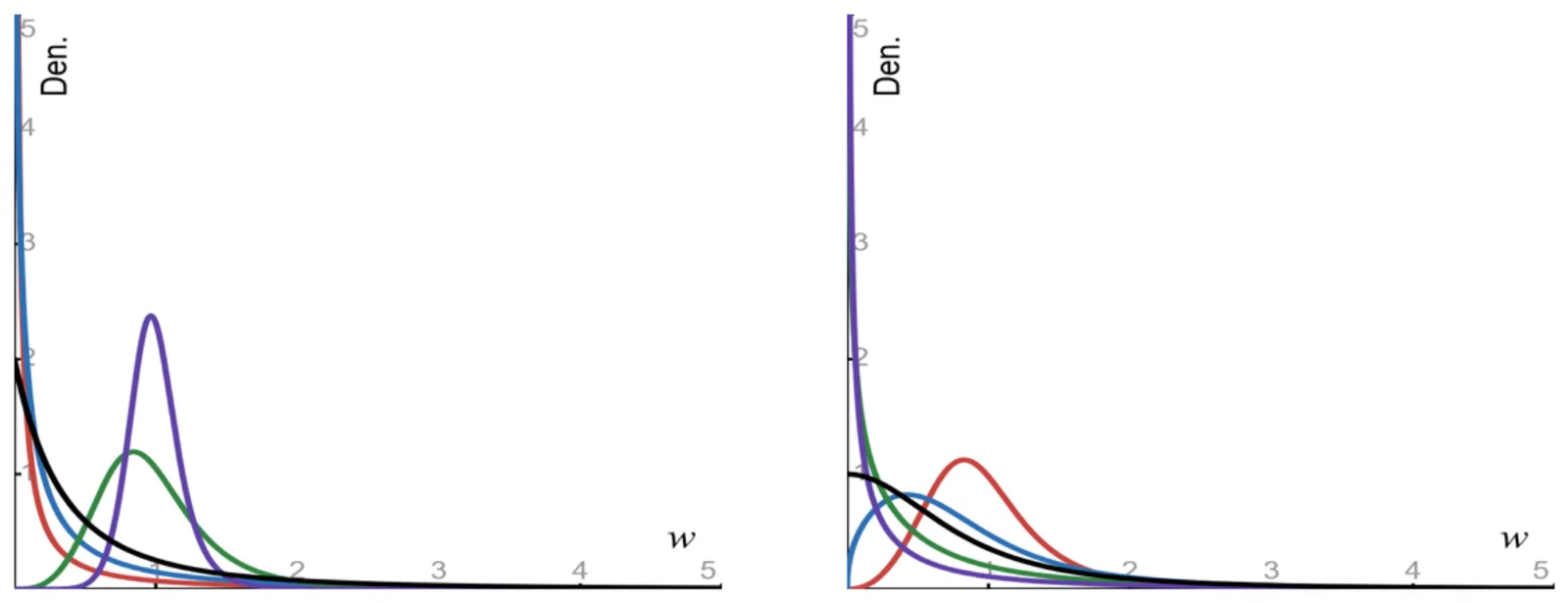
Examples of the density function of Equation (26). (**Left panel**) densities that correspond to the p-norm powers 0.3 (red), 0.4 (blue), 0.8 (green), 0.9 (purple), and 0.5 (black). (**Right panel**) densities that correspond to the p-norm powers 1.3 (red), 1.7 (blue), 3 (green), 5 (purple), and 2 (black).

The different shapes of the wealth density of Equation (26) are depicted in Figure 6. The classifications of the QLC wealth density are summarized in Table 5. These classifications address the following features of the QLC wealth density: the shape; the mode $w_{mod}$; the peak value $f\left(w_{mod}\right)$; and the variance Var[W] (which was addressed in Section 7.2).

**Phase transitions**. Section 5.3 and Section 6.1 described the QLC phase transition that takes place as the power *p* crosses the perfect-equality threshold p=1. This phase transition was illuminated from a Pareto-tails perspective (Section 5.3), and from a local-fractality perspective (Section 6.1).

This subsection unveils two more QLC phase transitions: one that takes place as the power *p* crosses the threshold $p=\frac{1}{2}$; and one that takes place as the power *p* crosses the threshold p=2. These phase transitions affect the features of the QLC wealth density of Equation (26) as follows.

On one ‘bank’ of this subsection’s phase transitions, the monotone-decreasing shape is that of an infinite slide; the mode is zero ($w_{mod}=0$); the peak value is infinite ($f\left(w_{mod}\right)=\infty$); and the variance is infinite (Var[W]=∞). The socioeconomic interpretation of this ‘bank’ is that of a polar society with two extreme classes: a vast class of poor Plebeians—which are reflected, statistically, by the zero mode and by the infinite peak value; and a tiny class of super-rich Patricians—which are reflected, statistically, by the infinite variance.

On the other ‘bank’ of this subsection’s phase transitions, the unimodal shape is that of a hill; the mode is positive ($0<w_{mod}<\infty$); the peak value is positive ($0<f\left(w_{mod}\right)<\infty$); and the variance is positive (0<Var[W]<∞). The socioeconomic interpretation of this ‘bank’ is that of a ‘normal’ society with three ‘normal’ classes: a small poor class—which is manifested by the hill’s left slope; a small rich class—which is manifested by the hill’s right slope; and a large middle class—which is manifested by the hill’s main bulk.

**Generalized-beta formulation**. Consider the probability density function

(27) $$f_{GB2}\left(w\right)=n\frac{w^{ca-1}}{{\left(1+w^{c}\right)}^{a+b}}=n\frac{w^{-cb-1}}{{\left(1+w^{-c}\right)}^{a+b}}$$

(0<w<∞), where *n* is a normalization constant; *a* and *b* are positive parameters; and *c* is a non-zero parameter. The density of Equation (27) manifests the “generalized beta distribution of the second kind” (GB2) statistical distribution [71,72,73,74,75,76,77]. Note that the GB2 distribution with parameters (a,b;−c) is identical to the GB2 distribution with parameters (b,a;c), i.e., switching the sign of the parameter *c* is equivalent to switching the parameters *a* and *b*.

Observing Equations (26) and (27), it follows that the latter ‘covers’ the former. Namely, the QLC wealth density of Equation (26) is a specific case of the GB2 density of Equation (27). Indeed—in terms of the GB2 parameters (a,b;c)—the QLC wealth density admits the following equivalent GB2 formulations: $\left(a,b;c\right)=\left(1,\frac{1}{p};-q\right)$ and $\left(a,b;c\right)=\left(\frac{1}{p},1;q\right)$.

The GB2 distribution has three parameters (the triplet (a,b;c)), whereas the wealth density of Equation (26) has only a single parameter (the power *p*). Thus, it is natural to expect the wealth statistics that are produced by the wealth density of Equation (26) to be less ‘rich’ than those produced by the GB2 density of Equation (27) [77]. Nonetheless, in essence, the wealth density of Equation (26) matches the ‘statistical riches’ of the GB2 density of Equation (27). So, parameter-wise, the QLC wealth statistics can be perceived as a ‘lean version’ of GB2 wealth statistics.

### 8.2. Skew Discussion

As is evident from the results of Section 8.1, the QLC wealth density $f\left(w\right)$ (0≤w≤∞) is skewed. This subsection will quantify the skewness of the QLC wealth density by comparing its median and mode to its mean. As set in the toolbox of Section 2.4, and as affirmed in Section 7.2: the QLC mean is one, μ=E[W]=1. The QLC median and the QLC mode are detailed below, and then the QLC skew is addressed.

**The median**. With regard to a human society of interest, the median $w_{med}$ is a value that splits the society’s population to two halves: one half comprises all the society members whose wealth is no larger than the median; and the other half comprises all the society members whose wealth is larger than the median. In terms of the society’s quantile function, $Q\left(u\right)$ (0≤u≤1), the median is the quantile of order half: $w_{med}=Q\left(\frac{1}{2}\right)$.

As derived in Section 5.3, the quantile function that corresponds to the QLC of Equation (14) is $Q\left(u\right)={\left(u^{-p}-1\right)}^{-1/q}$ (0≤u≤1), where p≠1. In turn, as a function of the positive power p≠1, the QLC median is

(28) $$w_{med}\left(p\right)={\left(2^{p}-1\right)}^{(1-p)/p}\;.$$

Note that Equation (28) extends continuously to the power p=1—for which the median is one, $w_{med}\left(1\right)=1$.

**Figure 7:**
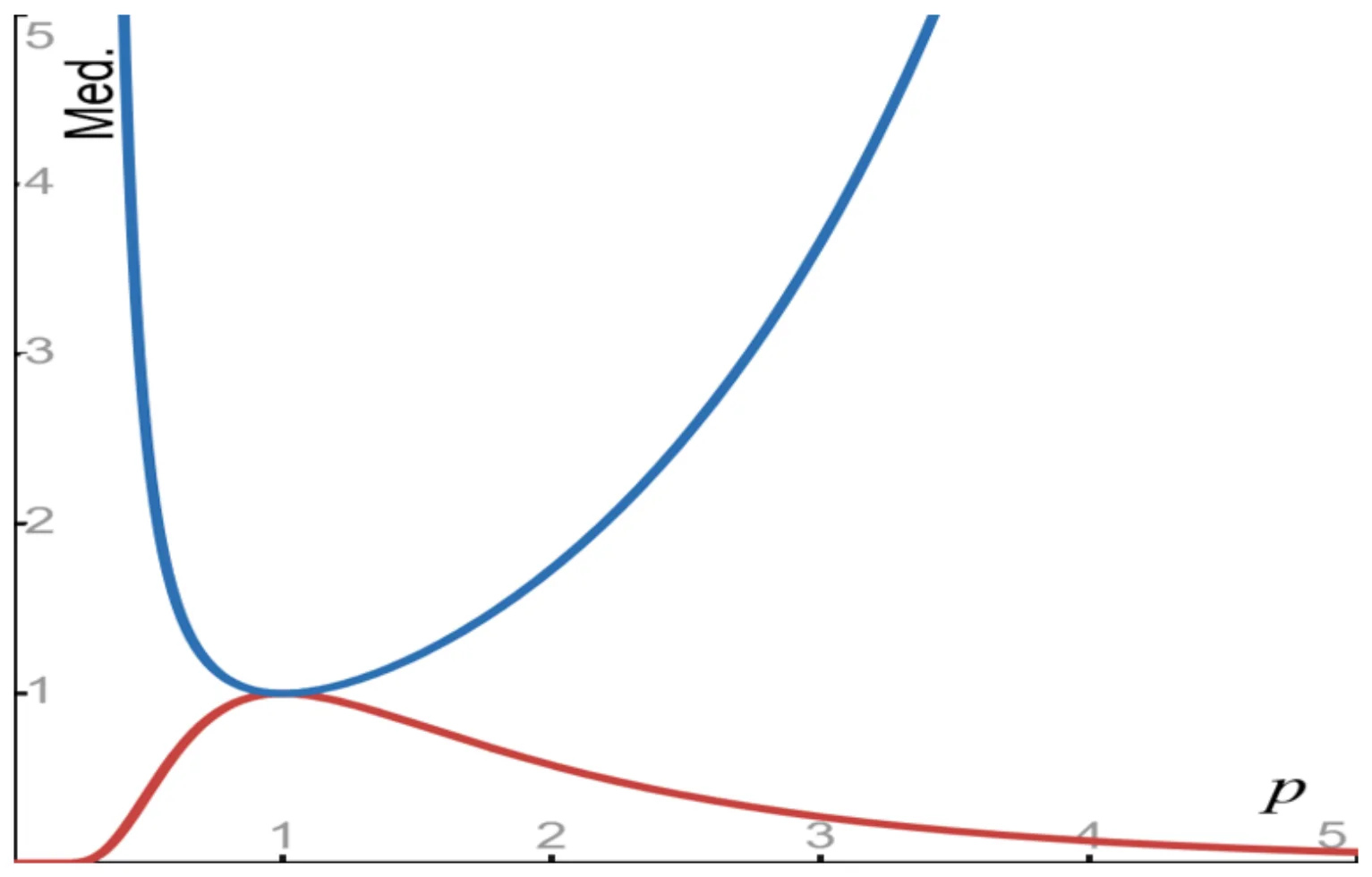
Medians as functions of the p-norm power. The median of Equation (28) (red), and the median of Equation (33) (blue).

As a function of the positive power *p*, the graph of the QLC median $w_{med}\left(p\right)$ is depicted in Figure 7. This graph has a unimodal shape that peaks at p=1. Specifically, the graph is monotone increasing over the range 0<p≤1 from zero ($\lim_{p\to 0}w_{med}\left(p\right)=0$) to one ($w_{med}\left(1\right)=1$), and monotone decreasing over the range 1≤p<∞ from one ($w_{med}\left(1\right)=1$) to zero ($\lim_{p\to \infty }w_{med}\left(p\right)=0$).

**The mode**. With regard to a wealth density of interest, $f\left(w\right)$ (0≤w≤∞), the mode is the most probable wealth value. Namely—implicitly assuming that the wealth density has a global maximum—the mode is the point at which the wealth density peaks: $w_{mod}$ $=\arg\max_{0\leq w\leq \infty }f\left(w\right)$. For the wealth density of Equation (26), the mode is as follows.

In the power ranges $0<p\leq \frac{1}{2}$ and 2≤p<∞ the mode is zero: $w_{mod}=0$. And in the power ranges $\frac{1}{2}<p<1$ and 1<p<2 the mode is the unique solution of $f^{\prime }\left(w\right)=0$. Differentiating the wealth density of Equation (26) and solving $f^{\prime }\left(w\right)=0$ yields the following result. As a function of the power *p*—where $\frac{1}{2}<p<1$ and 1<p<2—the QLC mode is

(29) $$w_{mod}\left(p\right)={\left(\frac{2p-1}{2-p}\right)}^{(1-p)/p}.\;$$

Note that Equation (29) extends continuously to the powers $p=\{\frac{1}{2},1,2\}$—for which the QLC mode values are $w_{mod}\left(\frac{1}{2}\right)=0=w_{mod}\left(2\right)$ and $w_{mod}\left(1\right)=1$.

**Figure 8:**
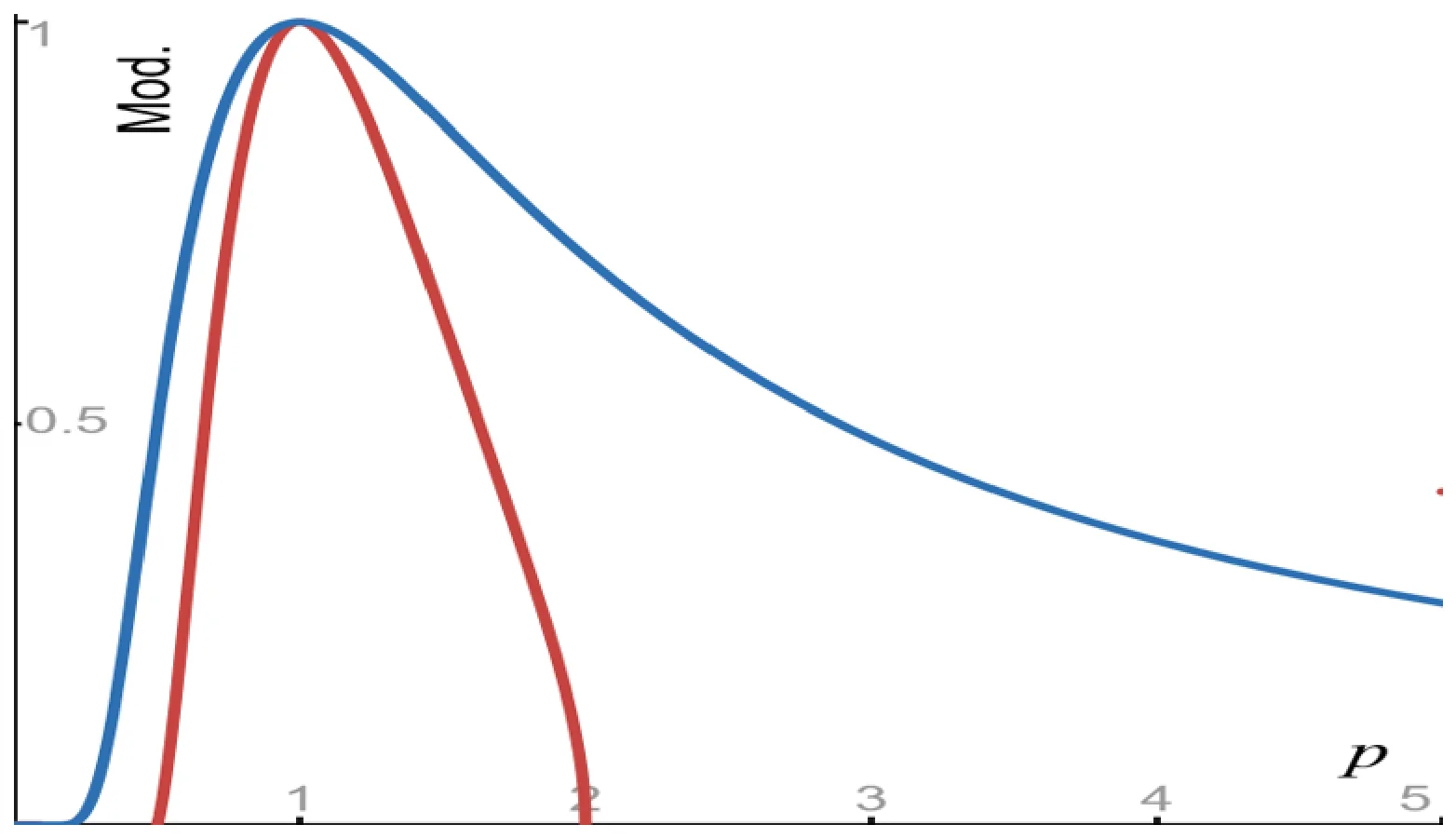
Modes as functions of the p-norm power. The mode of Equation (29) (red), and the mode of Equation (37) (blue).

As a function of the power $\frac{1}{2}\leq p\leq 2$, the graph of the QLC mode $w_{mod}\left(p\right)$ is depicted in Figure 8. This graph has a unimodal shape that peaks at p=1. Specifically, the graph is monotone increasing over the range $\frac{1}{2}\leq p\leq 1$ from zero ($w_{mod}\left(\frac{1}{2}\right)=0$) to one ($w_{mod}\left(1\right)=1$), and monotone decreasing over the range 1≤p≤2 from one ($w_{mod}\left(1\right)=1$) to zero ($w_{mod}\left(2\right)=0$).

**Skewness and inequality indices**. On the one hand, the QLC mean is one, μ=1. On the other hand, the peak values of the QLC median and of the QLC mode are one. Moreover, the QLC median and the QLC mode peak when—and only when—the power is one, p=1. And, due to the QLC Lorenz classification (see Table 3): the power p=1 characterizes the perfect-equality extreme.

Consequently: the QLC median-to-mean ratio is the median, $w_{med}\left(p\right)$$/\mu =w_{med}\left(p\right)$; the QLC mode-to-mean ratio is the mode, $w_{mod}\left(p\right)/\mu =w_{mod}\left(p\right)$; and the following conclusions hold. (**1**) These quantities take values in the unit-interval range: $0\leq w_{med}\left(p\right)\leq 1$ and $0\leq w_{mod}\left(p\right)\leq 1$. (**2**) These quantities yield the value one only in the perfect-equality extreme: $w_{med}\left(p\right)=1\Leftrightarrow p=1$ and $w_{mod}\left(p\right)=1\Leftrightarrow p=1$.

The large wealth of the rich society members cause the QLC mean μ to be greater than the QLC median and than the QLC mode. In turn, the following gaps are gauges of the wealth skewness: the gap between the QLC mean and median, $\mu -w_{med}\left(p\right)$; and the gap between the QLC mean and the mode, $\mu -w_{mod}\left(p\right)$.

Measuring these gaps relative to the QLC mean yields two QLC inequality indices (the notion of inequality indices was defined in the opening of Section 6.2). The QLC ‘median index’ is

(30) $$I_{med}\left(p\right)=\frac{\mu -w_{med}\left(p\right)}{\mu }=1-w_{med}\left(p\right)\;.$$

And the QLC ‘mode index’ is

(31) $$I_{mod}\left(p\right)=\frac{\mu -w_{mod}\left(p\right)}{\mu }=1-w_{mod}\left(p\right)\;.$$

These QLC inequality indices provide quantitative scores to the skewness of the QLC wealth density: the smaller the inequality indices, the smaller the skew, and the larger the inequality indices, the larger the skew.

### 8.3. Inequality Discussion

As defined in the opening of Section 6.2, inequality indices score the socioeconomic inequality of human societies. An inequality index yields a score *I* that takes values in the unit-interval range 0≤I≤1. The smaller the score, the more egalitarian the society. And the larger the score, the less egalitarian the society.

The minimal inequality score I=0 is assigned when—and only when—the human society under consideration is in the socioeconomic state of perfect equality. The maximal inequality score I=1 is assigned when—but not necessarily only when—the human society under consideration is in the socioeconomic state of perfect inequality. So, inequality indices always characterize the perfect-equality extreme, but they do not always characterize the perfect-inequality extreme.

Section 6.2 and Section 7.2, as well as Section 8.2, described several inequality indices. These inequality indices demonstrate the fact that there are many ways in which socioeconomic inequality can be gauged. In turn, each such gauge offers a different ‘angle’ to the measurement of socioeconomic inequality.

As summarized in Table 3—in the QLC context—the perfect-inequality extreme is attained when the positive power *p* is: either infinitely small, p→0; or infinitely large, p→∞. The inequality indices that characterize the perfect-inequality extreme are the following: the QLC Gini index of Equation (19); the QLC Pietra index of Equation (20); the QLC diameter index of Equation (21); and the QLC median index of Equation (30).

The inequality indices that do not characterize the perfect-inequality extreme are the following: the QLC variance index of Section 7.2, $I_{Var}\left(p\right)=\mathrm{Var}\left[W\right]/\left\{1+\mathrm{Var}\left[W\right]\right\}$; and the QLC mode index of Equation (31). These inequality indices are more sensitive to intense inequality, and they assign the maximal inequality score I=1 when the positive power *p* is either no larger than half, $p\leq \frac{1}{2}$, or no smaller than two, p≥2.

The specific powers $p=\frac{1}{2}$ and p=2 are of special importance. Indeed, these specific powers are the thresholds at which the phase transitions that were described in Section 8.1 occur. So, the QLC variance index and the QLC mode index echo these phase transitions: the two indices yield the maximal inequality score I=1 at the thresholds $p=\frac{1}{2}$ and p=2 (rather than at the limits p→0 and p→∞).

## 9. Quadrant Lorenz Curves: Alternative Wealth Sampling

Underpinning the QLC wealth statistics is the following ‘member sampling’ method. Sample at random a single member of the human society under consideration, and set *W* to be the wealth of the randomly sampled member.

An alternative sampling scheme is the following ‘Dollar sampling’ method. Sample at random a single dollar of the society’s overall wealth, and set $\tilde{W}$ to be the wealth of the society member to whom the randomly sampled dollar belongs.

The Dollar-sampling method is inclined towards the rich. Indeed, in the member-sampling method the realization of the random variable *W* is as likely to be the wealth of a rich society member as it is to be the wealth of a poor society member. In contrast, in the Dollar-sampling method the random variable $\tilde{W}$ is more likely to be the wealth of a rich society member.

To illustrate the inclination, consider a society with only two members: a poor member with personal wealth of 1 $; and a rich member with personal wealth of 10 $. On the one hand, in the member-sampling method the rich and poor outcomes occur with the same likelihood: $\mathrm{Pr}\left(W=1\right)=\frac{1}{2}$ and $\mathrm{Pr}\left(W=10\right)=\frac{1}{2}$. On the other hand, in the Dollar-sampling method the rich outcome is ten times more likely than the poor outcome: $\mathrm{Pr}\left(\tilde{W}=1\right)=\frac{1}{11}$ and $\mathrm{Pr}\left(\tilde{W}=10\right)=\frac{10}{11}$.

This section explores the shift from the member-sampling method to the Dollar-sampling method. Specifically, this section considers a human society whose wealth distribution is governed by the QLCs, and it investigates the statistics of the corresponding random variable $\tilde{W}$.

The statistics of the random variable $\tilde{W}$ are investigated via its distribution function (Section 9.1), and via its density function (Section 9.2). The investigation is followed by a comparison of the ‘*W* statistics’ vs. the ‘$\tilde{W}$ statistics’ (Section 9.3).

### 9.1. Distribution Function

The distribution function—be it bottom–up or top–down—of the random variable *W* is denoted $F\left(w\right)$ (0≤w≤∞). Analogously, denote by $\tilde{F}\left(w\right)$ (0≤w≤∞) the distribution function—also be it bottom–up or top–down—of the random variable $\tilde{W}$.

In effect, the Lorenz curves manifest a relationship between the two distribution functions. Indeed, $\tilde{F}\left(w\right)=L\left[F\left(w\right)\right]$ where [47,49,50]: the three functions involved—the distribution functions $F\left(w\right)$ and $\tilde{F}\left(w\right)$, and the Lorenz curve $y=L\left(x\right)$—are all either bottom–up or top–down.

Thus, substituting the distribution function of Equation (16) into the Lorenz curve of Equation (14) yields the following result. For p≠1, the distribution function of the random variable $\tilde{W}$ is

(32) $$\tilde{F}\left(w\right)=1-\frac{w^{-q/p}}{{\left(1+w^{-q}\right)}^{1/p}}=1-\frac{1}{{\left(1+w^{q}\right)}^{1/p}}$$

(0≤w≤∞).

As in the case of the distribution function of Equation (16): when the power is smaller than one (0<p<1) then the distribution function of Equation (32) is top–down; and when the power is larger than one (1<p<∞) then the distribution function of Equation (32) is bottom–up. The main implications that follow from the distribution function of Equation (32) are addressed below.

**Reciprocation**. Consider the reciprocal 1/R of a positive-valued random variable *R*. Denote by $G\left(r\right)$ (0≤r≤∞) the distribution function—be it bottom–up or top–down—of the random variable *R*. In terms of the distribution function $G\left(r\right)$, the distribution function of the reciprocal random variable 1/R admits the following formulation: 1−G(1/r) (0≤r≤∞). The distribution functions $G\left(r\right)$ and 1−G(1/r) are both either bottom–up or top–down.

Applying the transformation $G\left(r\right)\mapsto 1-G\left(1/r\right)$ to the distribution function of Equation (16) yields the distribution function of Equation (32). And, vice versa, applying the transformation $G\left(r\right)\mapsto 1-G\left(1/r\right)$ to the distribution function of Equation (32) yields the distribution function of Equation (16). Consequently, the following reciprocation relationship holds.

- The random variables *W* and $\tilde{W}$ are the reciprocals, in law, of each other: $W\overset{Law}{=}1/\tilde{W}$ and $\tilde{W}\overset{Law}{=}1/W$, where $\overset{Law}{=}$ denotes equality in law.

**Quantile function and median**. Inverting the distribution function of Equation (32) yields the corresponding quantile function: $\tilde{Q}\left(u\right)={\left[{\left(1-u\right)}^{-p}-1\right]}^{1/q}$ (0≤u≤1). In turn, setting $u=\frac{1}{2}$ in the quantile function yields the corresponding median: ${\tilde{w}}_{med}=\tilde{Q}\left(\frac{1}{2}\right)$. As a function of the positive power p≠1, the median is

(33) $${\tilde{w}}_{med}\left(p\right)={\left(2^{p}-1\right)}^{(p-1)/p}\;.$$

Note that Equation (33) extends continuously to the power p=1—for which the median is one, ${\tilde{w}}_{med}\left(1\right)=1$.

As a function of the positive power *p*, the graph of the median ${\tilde{w}}_{med}\left(p\right)$ is depicted in Figure 7. This graph has a V shape that bottoms out at p=1. Specifically, the graph is: monotone decreasing over the range 0<p≤1 from infinity ($\lim_{p\to 0}$${\tilde{w}}_{med}\left(p\right)=\infty$) to one (${\tilde{w}}_{med}\left(1\right)=1$); and monotone increasing over the range 1≤p<∞ from one (${\tilde{w}}_{med}\left(1\right)=1$) to infinity ($\lim_{p\to \infty }{\tilde{w}}_{med}\left(p\right)=\infty$).

It is evident from Equations (28) and (33) that: the medians $w_{med}\left(p\right)$ and ${\tilde{w}}_{med}\left(p\right)$ are the reciprocals of each other, i.e., $w_{med}\left(p\right)\cdot {\tilde{w}}_{med}\left(p\right)=1$. The reciprocation relationship of the medians is induced from the reciprocation relationship of the random variables *W* and $\tilde{W}$.

**Pareto tails**. As the distribution function of Equation (16), also the distribution function of Equation (32) displays Pareto tails. Indeed, in the top–down case (0<p<1) the Pareto tails are as follows.

- A left Pareto tail with exponent $\tilde{\alpha }=1/\left(1-p\right)$, i.e., at infinitesimally small wealth values the asymptotics are $1-\tilde{F}\left(w\right)∼$$w^{\tilde{\alpha }}$ (as w→0).
- A right Pareto tail with exponent $\tilde{\beta }=p/\left(1-p\right)$, i.e., at infinitely large wealth values the asymptotics are $\tilde{F}\left(w\right)∼1/w^{\tilde{\beta }}$ (as w→∞).

And in the bottom–up case (1<p<∞) the Pareto tails are as follows.

- A left Pareto tail with exponent $\tilde{\alpha }=p/\left(p-1\right)$, i.e., at infinitesimally small wealth values the asymptotics are $\tilde{F}\left(w\right)∼$$w^{\tilde{\alpha }}$ (as w→0).
- A right Pareto tail with exponent $\tilde{\beta }=1/\left(p-1\right)$, i.e., at infinitely large wealth values the asymptotics are $1-\tilde{F}\left(w\right)∼1/w^{\tilde{\beta }}$ (as w→∞).

The Pareto exponents—for both the top–down and bottom–up cases—admit the following formulations. As functions of the positive power p≠1, the left and the right Pareto exponents are, respectively,

(34) $$\tilde{\alpha }\left(p\right)=\frac{\max\{p,1\}}{\left|p-1\right|}\;\;\;\&\;\;\;\tilde{\beta }\left(p\right)=\frac{\min\{p,1\}}{\left|p-1\right|}\;.$$

It is evident from Equations (17) and (34) that the Pareto exponents of the random variable $\tilde{W}$ are the juxtaposition of the Pareto exponents of the random variable *W*, i.e., $\tilde{\alpha }\left(p\right)=\beta \left(p\right)$ and $\tilde{\beta }\left(p\right)=\alpha \left(p\right)$. The juxtaposition is induced from the reciprocation relationship of the random variables *W* and $\tilde{W}$. The graphs of the Pareto exponents of Equation (17) are depicted in Figure 4.

### 9.2. Density Function

The probability density function of the random variable *W* was denoted $f\left(w\right)$ (0≤w≤∞). Analogously, denote by $\tilde{f}\left(w\right)$ (0≤w≤∞) the probability density function of the random variable $\tilde{W}$. The relationship between the two density functions is [47]: $\tilde{f}\left(w\right)=wf\left(w\right)$. The main implications that follow from the density relationship are addressed below.

**Moments**. An immediate implication of the density relationship is the moments relationship $E\left[{\tilde{W}}^{m}\right]=E\left[W^{m+1}\right]$ (−∞<m<∞). In turn, Equation (25) further yields the moments relationship $E\left[{\tilde{W}}^{m}\right]=E\left[W^{-m}\right]$ (−∞<m<∞). Namely, the moment of order *m* of the random variable $\tilde{W}$ is the moment of order −m of the random variable *W*. This moments relationship is induced from the reciprocation relationship of Section 9.1.

Section 7.2 constructed a continuum of QLC moments inequality indices, $I_{m}\left(p\right)$ (0<m<∞), that admit the following formulations: $I_{m}\left(p\right)=1-1/E\left[W^{-m}\right]$ and $I_{m}\left(p\right)=1-1/E\left[W^{m+1}\right]$. The above moments relationships yield the following result. In terms of the random variable $\tilde{W}$, the QLC moments inequality indices admit the following re-formulations: $I_{m}\left(p\right)=1-1/E\left[{\tilde{W}}^{1-m}\right]$ and $I_{m}\left(p\right)=1-1/E\left[{\tilde{W}}^{m}\right]$.

**Mean**. Setting m=1 in the above moments relationships, and using the moments formula of Equation (23), yield the following result. The mean of the random variable $\tilde{W}$ is finite if and only if $\frac{1}{2}<p<2$, in which case

(35) $$E\left[\tilde{W}\right]=\frac{\Gamma \left(\frac{2}{p}-1\right)\Gamma \left(2-\frac{1}{p}\right)}{\Gamma \left(\frac{1}{p}\right)}\;.$$

Section 7.2 pointed out that setting m=1 in the QLC moments inequality indices yields the QLC variance index, $I_{Var}\left(p\right)=\mathrm{Var}\left[W\right]/\left\{1+\mathrm{Var}\left[W\right]\right\}$. In terms of the mean of the random variable $\tilde{W}$, the QLC variance index admits the re-formulation $I_{Var}\left(p\right)=1-1/E\left[\tilde{W}\right]$.

**Density function**. The density relationship and the wealth density of Equation (26) yields the following result. For p≠1, the density function of the random variable $\tilde{W}$ is

(36) $$\tilde{f}\left(w\right)=\frac{\left|q\right|}{p}\frac{w^{-q}}{{\left(1+w^{-q}\right)}^{(1/p)+1}}=\frac{\left|q\right|}{p}\frac{w^{q-1}}{{\left(1+w^{q}\right)}^{(1/p)+1}}$$

(0≤w≤∞).

An alternative way to derive the density function of Equation (36) is to differentiate the distribution function of Equation (32). The density function of Equation (36) peaks at a positive point ${\tilde{w}}_{mod}$$=\arg\max_{0\leq w\leq \infty }\tilde{f}\left(w\right)$, and its shape is unimodal: it is monotone increasing over the interval $\left[0,{\tilde{w}}_{mod}\right]$; and it is monotone decreasing over the ray $\left[{\tilde{w}}_{mod},\infty \right]$. The mode ${\tilde{w}}_{mod}$ is addressed below.

**Mode**. Differentiating the density function of Equation (36) and solving ${\tilde{f}}^{\prime }\left(w\right)=0$ yields the mode ${\tilde{w}}_{mod}$. As a function of the positive power p≠1, the mode is

(37) $${\tilde{w}}_{mod}\left(p\right)=p^{(1-p)/p}\;.$$

Note that Equation (37) extends continuously to the power p=1—for which the mode is one, ${\tilde{w}}_{mod}\left(1\right)=1$.

As a function of the positive power *p*, the graph of the mode ${\tilde{w}}_{mod}\left(p\right)$ is depicted in Figure 8. This graph has a unimodal shape that peaks at p=1. Specifically, the graph is: monotone increasing over the range 0<p≤1 from zero ($\lim_{p\to 0}{\tilde{w}}_{mod}\left(p\right)=0$) to one (${\tilde{w}}_{mod}\left(1\right)=1$); and monotone decreasing over the range 1≤p<∞ from one (${\tilde{w}}_{mod}\left(1\right)=1$) to zero ($\lim_{p\to \infty }{\tilde{w}}_{mod}\left(p\right)=0$).

**Generalized-beta formulation**. Section 8.1 showed that the QLC wealth density of Equation (26) is a specific case of the GB2 density of Equation (27). Specifically—in terms of the GB2 parameters (a,b;c)—the QLC wealth density admits the following equivalent GB2 formulations: $\left(a,b;c\right)=\left(1,\frac{1}{p};-q\right)$ and $\left(a,b;c\right)=\left(\frac{1}{p},1;q\right)$.

An analogous result holds for the density function of Equation (36) as well: this density is also a specific case of the GB2 density of Equation (27). Indeed—in terms of the GB2 parameters (a,b;c)—the density function of Equation (36) admits the following equivalent GB2 formulations: $\left(a,b;c\right)=\left(1,\frac{1}{p};q\right)$ and $\left(a,b;c\right)=\left(\frac{1}{p},1;-q\right)$.

Observing the GB2 formulations of the density functions of Equations (26) and (36), note that switching from the formulation of the former to that of the latter is equivalent to switching the sign of the GB2 parameter *c*. This ‘switching equivalence’ is induced from the reciprocation relationship of Section 9.1.

### 9.3. Comparison

On the one hand, the member-sampling method generates—via the random variable *W*—wealth statistics that were analyzed in the sections above. On the other hand, the Dollar-sampling method generates—via the random variable $\tilde{W}$—wealth statistics that were analyzed in this section.

The two sampling methods yield the same statistics when—and only when—the distribution of wealth is perfectly equal, i.e., all society members have the same wealth. As noted above, perfect equality is characterized by the power p=1—in which case both random variables are one, Pr(W=1)=1 and $\mathrm{Pr}(\tilde{W}=1)=1$.

When p≠1 then the ‘*W* statistics’ and the ‘$\tilde{W}$ statistics’ differ. This subsection compares the two wealth statistics. With regard to the power ranges $0<p\leq \frac{1}{2}$ and 2≤p<∞, the comparison is summarized in Table 6.

**Densities**. The density function f(w) is unimodal when the power is in the range $\frac{1}{2}<p<2$, and is monotone decreasing otherwise. In contrast, the density function $\tilde{f}\left(w\right)$ is always unimodal.

**Pareto tails**. Both the distribution functions $F\left(w\right)$ and $\tilde{F}\left(w\right)$ have left and right Pareto tails. In terms of the Pareto-exponents formulations of Equations (17) and (34): the left and right Pareto exponents of $F\left(w\right)$ are, respectively, the right and left Pareto exponents of $\tilde{F}\left(w\right)$.

**Means**. The mean of the random variable *W* is always one. In contrast, the mean of the random variable $\tilde{W}$ is a function of the power *p*: it is finite when the power is in the range $\frac{1}{2}<p<2$, and it is infinite otherwise. In terms of the QLC variance index (of Section 7.2), the means’ ratio admits the following formulation: $E\left[W\right]/E\left[\tilde{W}\right]=1-I_{Var}\left(p\right)$.

**Medians**. The medians $w_{med}\left(p\right)$ and ${\tilde{w}}_{med}\left(p\right)$ are functions of the power *p*, and they are the reciprocals of each other: $w_{med}\left(p\right)\cdot {\tilde{w}}_{med}\left(p\right)=1$. Consequently, the medians have antithetical shapes: the median $w_{med}\left(p\right)$ has a unimodal shape with peak value $w_{med}\left(1\right)=1$; and the median ${\tilde{w}}_{med}\left(p\right)$ has a V shape with bottom value ${\tilde{w}}_{med}\left(1\right)=1$.

**Modes**. The modes $w_{mod}\left(p\right)$ and ${\tilde{w}}_{mod}\left(p\right)$ are functions of the power *p*. Both these modes have a unimodal shape with peak values $w_{mod}\left(1\right)=1$ and ${\tilde{w}}_{mod}\left(1\right)=1$.

## 10. Conclusions

Lorenz curves [38,39,40,41] offer a socioeconomic representation of the statistics of positive-valued random variables. Lorenz curves reside in the unit square, and they have convex and concave shapes. In effect, Lorenz curves map geometric shapes to statistical distributions [49,50].

Arc Lorenz curves [51] have shapes that are arcs of Euclidean circles. Power Lorenz curves [42] have power-law shapes that characterize socioeconomic fractality: rich fractality, which represents Pareto statistics of ‘type I’; and poor fractality, which represents inverted Pareto statistics of ‘type I’. Arc Lorenz curves and power Lorenz curves, as well as the statistical distributions they represent, have upsides (merits) and downsides (limitations).

‘Elevating’ from the Euclidean norm to the non-Euclidean *p*-norm, this paper devised Quadrant Lorenz Curves (QLCs): Lorenz curves whose shapes are that of the symmetric quarters of the *p*-norm unit circle. Deeming arc Lorenz curves to be a ‘father DNA’, and deeming power Lorenz curves to be a ‘mother DNA’: QLCs are the ‘offspring’ whose recombined DNA inherits the upsides—but not the downsides—of both its ‘parents’.

On the one hand: QLCs have a single parameter, the positive power of the *p*-norm; and their shapes have a clear geometric meaning. On the other hand, QLCs produce an assortment of socioeconomic and statistical features. These features were comprehensively explored in Section 5, Section 6, Section 7, Section 8 and Section 9 above, and some of these features are summarized in Table 3, Table 4, Table 5 and Table 6. The exploration included a multi-faceted socioeconomic-inequality analysis—via a host of inequality indices—of QLCs: see Section 6.2, Section 7.2, Section 8.2 and Section 8.3.

As noted in the introduction, the QLCs manifest a novel class of ‘fractal’ statistical distributions. Importantly, the socioeconomic and statistical features of the QLCs are unmatched by any other class of fractal statistical distributions (which is known to date).

As explained in Section 8.1, the statistics that QLCs represent are a subset of the following statistics: “generalized beta of the second kind” (GB2) [71,72,73,74,75,76,77]; excluding scale, GB2 statistics have three parameters. GB2 statistics are a well-established model for income distributions, and recent examples of research involving these statistics include: rethinking GB2 [78], population growth [79], open-source repositories [80], goodness-of-fit [81], and heart rate and blood pressure [82].

In turn, GB2 statistics are a subset of general “double Pareto” statistics [83,84]: size distributions that display both left and right Pareto tails. Double-Pareto statistics appear across the sciences, e.g., in economics [84,85,86], demography [87,88], geophysics [89,90,91], and networks [92,93,94]. Recent examples of research involving these statistics include: fund returns [95], autoregression models [96], uniform observation times [97,98], and Levy flights [99].

As established recently in [77], GB2 statistics are a particular outcome of a double-Pareto ‘supermodel’: a general diffusion framework for the generation of double-Pareto models. This diffusion framework has a ‘core’ that has the capacity to accommodate as many parameters as desired, and different cores generate different double-Pareto models.

All models that are generated by the diffusion framework have a universal approximation [77]: the double-Pareto-lognormal (DPLN) model [100,101]. Also generated by the diffusion framework, the DPLN model ‘stitches’ together the three following statistics: inverted Pareto of ‘type I’ for small sizes; Pareto of ‘type I’ for large sizes; and lognormal for intermediate sizes. The ‘stitching’ takes place at two bars [77]: a ‘poverty bar’ that separates the small sizes from the intermediate sizes; and a ‘riches bar’ that separates the intermediate sizes from the large sizes.

The QLC model is a special case of the GB2 model—which, in turn, is generated by the diffusion framework. So, in particular, the DPLN approximation holds for the QLC model. From the perspective of the aforementioned mother-DNA analogy, this fact ‘closes the circle’. Indeed, with regard to the DPLN approximation of the QLC model: the Pareto parts of this approximation are in perfect match with the mother-DNA—the power Lorenz curves.

Setting off from the GB2 statistics, one can go—parameter-wise, and in the realm of double-Pareto statistics—either up or down. Going up yields the diffusion framework which, parameter-wise, is abundant (accomodating as many parameters as desired). Going down yields the QLC model which, parameter-wise, is as lean as possible (having only a single parameter).

Remarkably so, the lean QLC model ‘punches way above its weight’. Indeed, with its single parameter—the positive power of the *p*-norm—the QLC model generates an assortment of socioeconomic and statistical features. And, in essence, the features of the QLC model are almost as ‘rich’ as the features of the diffusion framework.

The drawback of the QLC model relative to the diffusion framework is the following. The fact that the QLC model has a single parameter implies that its left and right Pareto tails are coupled—whereas the left and right Pareto tails of the diffusion framework are not. Apart from this drawback, the QLC model is a ‘microcosm’ of the diffusion framework. Thus, the QLC model provides a rather impressive ‘return on investment’—the ‘investment’ being the parameters, and the ‘return’ being the socioeconomic and statistical features.

Metaphorically, envisage double-Pareto statistics as a geographical region. The region includes several powers: the GB2 model, the DPLN model, and the diffusion framework ‘superpower’. Geographically and geopolitically, the region and its powers are well known and well studied. In this metaphorical context, this paper discovered and charted a hidden valley with a city-state—the QLC model—which, to date, were unnoticed and unexplored. Moreover, the paper investigated the newly discovered valley and city-state, and showed that they are geographic and geopolitical ‘microcosms’ of the region and of its superpower.

## Figures and Tables

**Table 1:** The PLC of Equation (6). The curve is parametrized by the positive power *p*. Different values of the parameter (left column) determine: the curve’s Lorenz classification (middle column); and the classification of the corresponding socioeconomic states.

Parameter	Lorenz Curve	Socioeconomic State
p→0	top down	perfect inequality
0<p<1	top down	richfractalityexp.p
p=1	diagonal line	perfect equality
1<p<∞	bottom up	poorfractalityexp.p
p→∞	bottom up	perfect inequality

**Table 2:** The rich-fractality and the poor-fractality socioeconomic states. These states are governed by the wealth distribution function $F\left(w\right)$ of Equation (7). The table’s rows specify: the ranges of the power parameter *p*; the bounds of the wealth values; and the Pareto tails of the wealth statistics.

	Rich Fractality	Poor Fractality
Parameter range	0<p<1	1<p<∞
Lower bound	$w_{\min}=p$	$w_{\min}=0$
Upper bound	$w_{\max}=\infty$	$w_{\max}=p$
Pareto tail	$F\left(w\right)\underset{w\to \infty }{∼}1/w^{\beta }$	$F\left(w\right)\underset{w\to 0}{∼}w^{\alpha }$
Pareto exponent	$\beta =\frac{1}{1-p}$	$\alpha =\frac{1}{p-1}$

**Table 3:** The QLC of Equation (14). The curve is parametrized by the positive power *p*. Different values of the parameter (left column) determine: the curve’s Lorenz classification (middle column); and the corresponding socioeconomic behaviors (right column). The notions of local fractality (noted in right column) are explained and discussed in Section 6 below.

Parameter	Lorenz Curve	Socioeconomic Behaviors
p→0	top–down	perfect inequality
0<p<1	top–down	local rich fractality exp. *p*
		local poor fractality exp. 1/p
p=1	diagonal line	perfect equality
1<p<∞	bottom–up	local rich fractality exp. 1/p
		local poor fractality exp. *p*
p→∞	bottom–up	perfect inequality

**Table 4:** The QLC wealth statistics. These wealth statistics are governed by the distribution function $F\left(w\right)$ of Equation (16), which is parametrized by the positive power p≠1. The table’s middle and right columns address the two different ranges of the parameter—below and above the perfect-equality threshold p=1. The table’s rows specify: the bounds of the wealth values; and the Pareto tails of the wealth statistics.

	0<p<1	1<p<∞
Wealth bounds	$\begin{array}{c} w_{\min}=0\\ w_{\max}=\infty \end{array}$	$\begin{array}{c} w_{\min}=0\\ w_{\max}=\infty \end{array}$
Left Pareto tail	$1-F\left(w\right)\underset{w\to 0}{∼}w^{\alpha }$	$F\left(w\right)\underset{w\to 0}{∼}w^{\alpha }$
Left Pareto exp.	$\alpha =\frac{p}{1-p}$	$\alpha =\frac{1}{p-1}$
Right Pareto tail	$F\left(w\right)\underset{w\to \infty }{∼}1/w^{\beta }$	$1-F\left(w\right)\underset{w\to \infty }{∼}1/w^{\beta }$
Right Pareto exp.	$\beta =\frac{1}{1-p}$	$\beta =\frac{p}{p-1}$

**Table 5:** The QLC wealth density $f\left(w\right)$ (0≤w≤∞). This density is parametrized by the positive power *p*. Different values of the parameter (left column) determine the density’s features: the shape; the mode $w_{mod}$; the peak value $f\left(w_{mod}\right)$; and the variance Var[W]. In the table’s cells the sign “+” denotes positive values.

Parameter	Shape	Mode $w_{\mathrm{mod}}=$	Peak $f\left(w_{\mathrm{mod}}\right)=$	Variance Var[W]=
$0<p<\frac{1}{2}$	monotone	0	*∞*	*∞*
$p=\frac{1}{2}$	monotone	0	+	*∞*
$\frac{1}{2}<p<1$	unimodal	+	+	+
p=1	singular	1	*∞*	0
1<p<2	unimodal	+	+	+
p=2	monotone	0	+	*∞*
2<p<∞	monotone	0	*∞*	*∞*

**Table 6:** Summary of the ‘*W* statistics’ vs. the ‘$\tilde{W}$ statistics’ in the power ranges $0<p\leq \frac{1}{2}$ and 2≤p<∞. In the bottom rows of the table: the sign ↘ means a decay to zero as p→0 and p→∞; and the sign ↗ means a growth to infinity as p→0 and p→∞.

	*W* Statistics	$\tilde{W}$ Statistics
**Density shape**	monotone	unimodal
**Left Pareto exp.**	$\alpha =\frac{p}{1-p}$	$\tilde{\alpha }=\frac{1}{p-1}$
**Right Pareto exp.**	$\beta =\frac{1}{1-p}$	$\tilde{\beta }=\frac{p}{p-1}$
**Mean**	$E\left[W\right]=1$	$E[\tilde{W}]=\infty$
**Median limits**	$w_{med}\left(p\right)↘0$	${\tilde{w}}_{med}\left(p\right)↗\infty$
**Mode limits**	$w_{mod}\left(p\right)↘0$	${\tilde{w}}_{mod}\left(p\right)↘0$

## Data Availability

No new data were created or analyzed in this study.
